# Polydatin-Mediated Inhibition of HSP90α Disrupts NLRP3 Complexes and Alleviates Acute Pancreatitis

**DOI:** 10.34133/research.0551

**Published:** 2024-12-17

**Authors:** Jiashu Yang, Chenyang Jiao, Nannan Liu, Wen Liu, Yueyao Wang, Ying Pan, Lingdong Kong, Wenjie Guo, Qiang Xu

**Affiliations:** ^1^ State Key Laboratory of Pharmaceutical Biotechnology, Department of Gastroenterology, Nanjing Drum Tower Hospital, School of Life Sciences, Nanjing University, Nanjing, China.; ^2^ School of Pharmacy, Nanjing University of Chinese Medicine, Nanjing, China.

## Abstract

The NLRP3 inflammasome plays a critical role in various inflammatory conditions. However, despite extensive research in targeted drug development for NLRP3, including MCC950, clinical success remains elusive. Here, we discovered that the activated NLRP3 inflammasome complex (disc-NLRP3) and the activating mutation L351P exhibited resistance to MCC950. Through investigations using the small-molecule compound polydatin, HSP90α was found to stabilize both the resting (cage-NLRP3) and activated state (disc-NLRP3) of NLRP3 complexes, sustaining its activation. Our mechanistic studies revealed that polydatin specifically targets HSP90α, binding to it directly and subsequently interfering with the HSP90α-NLRP3 interaction. This disruption leads to the dissipation of cage-NLRP3, disc-NLRP3 complexes and NLRP3 L351P. Importantly, genetic and pharmacological inactivation of HSP90α effectively reduced NLRP3 inflammasome activation and alleviated cerulein-induced acute pancreatitis. These therapeutic effects highlight the clinical potential of HSP90α inhibition. Our findings demonstrate that HSP90α is crucial for the stability of both the resting and activated states of the NLRP3 inflammasome during its sustained activation, and targeting HSP90α represents a promising therapeutic strategy for diseases driven by the NLRP3 inflammasome.

## Introduction

Inflammasomes are multi-protein complexes formed by cytoplasmic pattern recognition receptors. These receptors play a pivotal role in detecting both pathogen-associated molecular patterns and host-derived damage-associated molecular patterns [1]. As key components of the innate immune system, these complexes orchestrate innate immune responses, aiding in host defense against infections as well as in the regulation of autoimmune and inflammatory disorders [2–4]. NACHT, LRR, and PYD domain-containing protein 3 (NLRP3) comprises 3 domains: a C-terminal leucine-rich repeat (LRR) domain, nucleotide-binding domain with adenosine triphosphatase (ATPase) activity (NACHT domain), and an N-terminal pyrin (PYD) domain [5,6]. Structurally and functionally, the abnormal activation of the NLRP3 inflammasome has been linked to the development of several diseases, including gouty arthritis, pancreatitis, multiple sclerosis, and Alzheimer’s disease [7,8]. Therefore, the pharmacological inhibition of NLRP3 represents a potentially effective treatment strategy. Although various NLRP3 inflammasome inhibitors have shown promising preventive and therapeutic effects in animal models of associated diseases, several inhibitors have progressed to phase II clinical trials. For instance, MCC950, also referred to as CRID3, has advanced to phase II trials for rheumatoid arthritis. However, the trial revealed elevated levels of alanine aminotransferase and aspartate aminotransferase in the serum, leading to project termination [6]. Twenty years after the discovery of CP-456773, Genentech relaunched the clinical trial, but later discovered that the compound could accumulate in the kidneys due to pH-dependent solubility and declared the project a failure [9]. OLT1177, another NLRP3 inhibitor with potential clinical application, is currently in phase II clinical trials. However, current research indicates that OLT1177 is likely to face setbacks in clinical trials similar to MCC950, primarily because of the lack of a robust dose–response relationship at high concentrations [10]. Hence, there persists a pressing demand for the development of novel and more effective NLRP3 inhibitors. Generally, activation of the NLRP3 inflammasome involves 2 signals. The first step, known as the priming signal, is typically induced by microbial lipopolysaccharide (LPS). The second step, referred to as the activating signal, is commonly triggered by adenosine triphosphate (ATP), monosodium urate (MSU), or nigericin [11]. Recent advances in inflammasome research have revealed crucial insights into both the structural organization and regulatory mechanisms of endogenous NLRP3 under physiological conditions. Specifically, endogenous full-length NLRP3 forms membrane-localized double-ring cage-like structures that remain inactive in an autoinhibitory state, contributing to intracellular inflammasome homeostasis [12]. This structural configuration serves as a critical checkpoint in preventing inappropriate inflammasome activation. The cage-like structure is crucial for converting an NLRP3 activation stimulus into a trans-Golgi network (TGN) dispersion, which is essential for NLRP3 activation [11,13]. Following this, the scattered TGN vesicles interact with NIMA-related kinase 7 (NEK7), leading to the formation of active puncta of the NLRP3 inflammasome [14,15]. Finally, the disc-shaped active NLRP3 oligomer complexing NEK7 and ASC is formed [16].

In our investigation, we showed that the activated NLRP3 inflammasome complex (disc-NLRP3) and the activating mutation NLRP3 L351P were found to be resistant to MCC950. Specifically, MCC950 was not able to inhibit the function of the activated NLRP3 inflammasome complexes. HSP90α was found to stabilize both the resting (cage-NLRP3) and activated (disc-NLRP3) states of NLRP3 complexes, sustaining their activation, which relies on its ATPase activity. This stabilizing effect appears to be essential for maintaining the functional integrity of both conformational states. Pharmacological inactivation of HSP90α led to the disruption of cage-NLRP3 and disc-NLRP3 complexes and NLRP3 L351P, distinct from that of MCC950. These findings highlight a novel mechanism of NLRP3 regulation distinct from conventional inhibitors. Collectively, our study proposes a new strategy for regulating the NLRP3 inflammasome by disrupting the structural integrity of the NLRP3 inflammasome complex. Furthermore, polydatin has been identified as a promising therapeutic agent that, by targeting different states of the NLRP3 inflammasome complex, shows potential in treating acute pancreatitis (AP) and other NLRP3-related inflammatory diseases.

## Results

### MCC950 fails to inhibit the activated complexes of the NLRP3 inflammasome and the gain-of-function mutation NLRP3 L351P

**Fig. 1.**
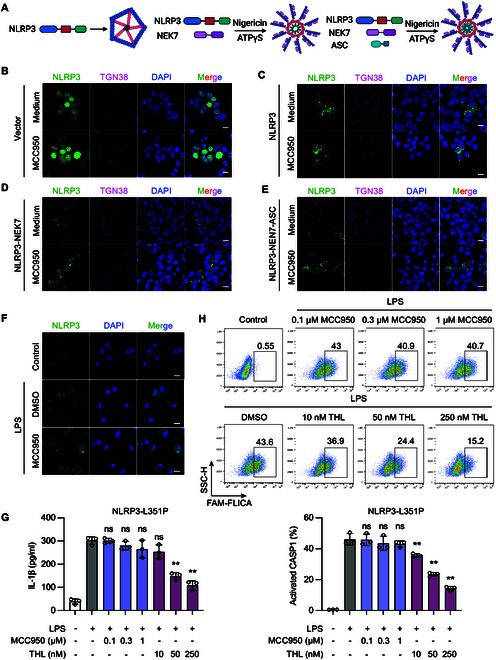
The activation form NLRP3 inflammasome complex shows resistance to MCC950. (A) Cage-NLRP3, NLRP3-NEK7, and NLRP3-NEK7-ASC complexes were re-constituted in HEK293T cells. For cage-NLRP3, HEK293T cells were transfected with GFP-NLRP3 only; for the NLRP3-NEK7 complex, HEK293T cells cotransfected with GFP-NLRP3 and HA-NEK7 were stimulated with nigericin, and then ATPγS and MgCl_2_ were added to lock NLRP3 in an active conformation. For NLRP3-NEK7-ASC complex, HEK293T cells cotransfected with GFP-NLRP3, HA-NEK7, and ASC^PYD^ were stimulated with nigericin, and then ATPγS and MgCl_2_ were added. (B) GFP-tagged vector (C) Cage-NLRP3, (D) NLRP3-NEK7, and (E) NLRP3-NEK7-ASC complexes were reproduced in HEK293T cells. The effect of MCC950 on 3 stages of NLRP3 complexes was observed by confocal microscopy. (F) BMDMs stimulated with 100 ng/ml LPS were treated with 1 μM MCC950 for 1 h. NLRP3 oligomer formation was examined using IF. (G and H) NLRP3^lyz−/−^ BMDMs were recombined with mouse NLRP3 L351P mutant (L351P-BMDM). L351P-BMDMs were treated with indicated concentrations of MCC950 or THL followed by 100 ng/ml LPS for 3 h. (G) IL-1β in the supernatant was determined by ELISA. (H) CASP1 activation was determined by FAM-FLICA staining. Scale bar, 10 μm. Data are presented as mean ± SEM of 3 independent experiments.

First, we reconstituted cage-NLRP3, NEK7-NLRP3, and NEK7-NLRP3-ASC complexes in HEK293T cells. Specifically, we transfected cells with green fluorescent protein (GFP)-tagged full-length NLRP3 or cotransfected them with GFP-NLRP3 and hemagglutinin (HA)-NEK7 with or without ASC^PYD^. The cells were then exposed to nigericin and the ATP analog adenosine 5′-O-(3-thio)triphosphate (ATPγS) in the presence of MgCl₂ to promote stabilization of NLRP3 in its active conformation (Fig. 1A). The reconstituted cage-NLRP3 construct exhibited substantial aggregation, manifesting as large, bright spots under microscopy. Cotransfection with NEK7 and NLRP3 resulted in the gradual dispersion of these large spots into smaller ones. Ultimately, the NLRP3-ASC-NEK7 complex showed a distribution characterized by small bright spots in the cytoplasm (Fig. S1A).

The engagement of MCC950 with residues from 5 distinct subdomains within the NLRP3 cleft stabilizes their conformational alignment, locking the structure and impairing its ability to undergo conformational changes, which inactivates the NLRP3 inflammasome. However, the formation of a cage-like decamer structure by NLRP3 creates spatial constraints that prevent MCC950 from penetrating and binding effectively, posing a challenge in targeting NLRP3 after its assembly [17]. To examine whether the assembled NLRP3 complex hinders the penetration and inhibitory effect of MCC950, we reconstructed cage-NLRP3, NLRP3-NEK7, and NLRP3-NEK7-ASC complexes in HEK293T cells. We observed that MCC950 was ineffective against the fully assembled NLRP3 complex (Fig. 1B to E). This observation suggests that MCC950’s effectiveness was markedly limited, failing to exert a substantial inhibitory effect on the formation of NLRP3 complexes in cells under inflammatory conditions. Likewise, upon stimulation of bone marrow–derived macrophages (BMDMs) with LPS, we observed an increase in the expression of NLRP3 accompanied by the formation of structured aggregates. Subsequent treatment with MCC950 failed to visibly affect these preexisting NLRP3 aggregates, which is consistent with earlier findings (Fig. 1F) [18]*.* In summary, MCC950 was ineffective against the fully assembled NLRP3 complex.

Gain-of-function mutations within the NACHT domain of NLRP3 and neighboring regions are linked to 3 autosomal dominant periodic fever disorders collectively classified as cryopyrin-associated periodic syndromes (CAPS) [19]. To further investigate the efficacy of MCC950 against NLRP3 gain-of-function mutations, we utilized BMDMs from NLRP3 knockout (KO) mice and introduced mouse NLRP3 mutations to assess the efficacy of MCC950 in preventing activation of the NLRP3 inflammasome triggered by gain-of-function mutations. Our research indicates that, unlike thiolutin (THL), which is another small molecule that significantly inhibits the activation of CAPS-related NLRP3 mutants [20], MCC950 is ineffective in reducing the activity of the CAPS-related NLRP3 mutant L351P. This is evidenced by its failure to decrease interleukin-1β (IL-1β) secretion and to mature CASP1 (Fig. 1G and H). These findings highlight the limitations of MCC950 in addressing NLRP3-mediated inflammation, particularly in the context of gain-of-function mutations. In summary, conventional NLRP3 inhibitors such as MCC950 exhibit limitations in suppressing inflammation once NLRP3 is activated. Hence, there is a critical need to investigate innovative strategies to achieve effective anti-inflammatory outcomes.

### Polydatin potentially inhibits the formation of cage-NLRP3

**Fig. 2.**
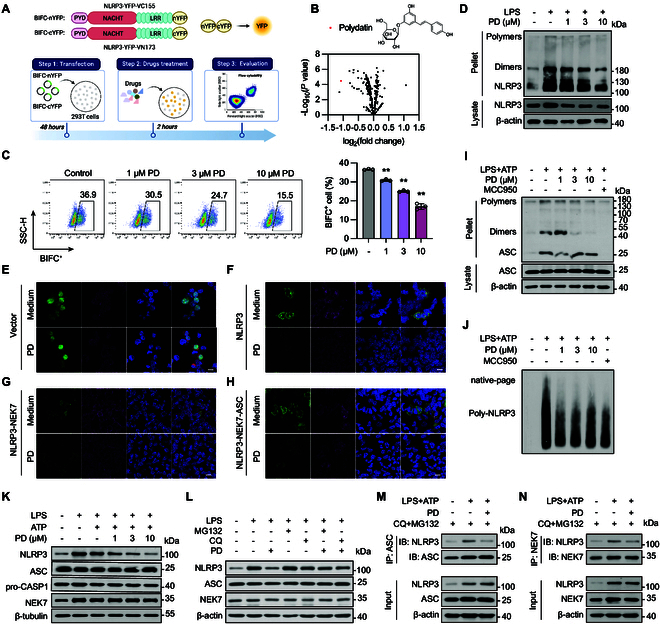
Polydatin is a potential natural product that inhibited the formation of cage-NLRP3 and disc-NLRP3. (A) Schematic representation of screening natural products targeting cage-NLRP3 using flow cytometry. (B) HEK293T cells cotransfected with BIFC-nYFP-NLRP3 and BIFC-cYFP-NLRP3 were treated with different natural products, and the BIFC^+^ cells were determined by flow cytometry. The chemical structure of polydatin was shown. (C) HEK293T cells cotransfected with BIFC-nYFP-NLRP3 and BIFC-cYFP-NLRP3 were treated with indicated concentrations of polydatin, and the BIFC^+^ cells were determined by flow cytometry. (D) BMDMs were stimulated with 100 ng/ml LPS for 3 h, followed by indicated concentrations of polydatin treatment for 1 h. Oligomerization of NLRP3 in the Nonidet P40-insoluble pellet and NLRP3 in cell lysates were examined using Western blotting. (E) GFP-tagged vector. (F) Cage-NLRP3, (G) NLRP3-NEK7, and (H) NLRP3-NEK7-ASC complexes were reproduced in HEK293T cells. The effect of polydatin on 3 stages of NLRP3 complexes was observed by confocal microscopy. (I and J) LPS-primed BMDMs were treated with polydatin for 1 h, followed by ATP stimulation for 30 min. (I) ASC in cell lysates and oligomerization of ASC in the Nonidet P40-insoluble pellet were examined via Western blotting. (J) Oligomerization of NLRP3 was analyzed by blue native PAGE. (K) BMDMs were exposed to 100 ng/ml LPS for 3 h, followed by treatment with specified concentrations of polydatin for 1 h, and subsequently stimulated with 5 mM ATP for another 1 h. Western blotting analysis of NLRP3, ASC, NEK7, and pro-CASP1. (L) BMDMs were treated with 20 μM MG132 or 30 μM CQ for 2 h, and then 100 ng/ml LPS for 3 h followed by 10 μM polydatin treatment for 1 h. The expression of NLRP3 was determined by Western blot. (M and N) BMDMs were pretreated with CQ and MG132, followed by LPS and ATP stimulation, and an endogenous immunoprecipitation (IP) was performed using (M) ASC antibody or (N) NEK7 antibody, with or without polydatin treatment. Scale bar, 10 μm. Data are presented as mean ± SEM of 3 independent experiments. *P < 0.05, **P < 0.01.

To identify drugs capable of effectively inhibiting the NLRP3 complex, we utilized bimolecular fluorescence complementation (BiFC) assay to screen compounds from a natural product library that could hinder the formation of the cage-NLRP3 complex (Fig. 2A). Notably, polydatin demonstrated a potent inhibitory effect on the formation of cage-NLRP3 (Fig. 2B). To further confirm this inhibitory effect, cells were exposed to different concentrations of polydatin. The BiFC signals exhibited a dose-dependent reduction, strongly suggesting that polydatin effectively interferes with the formation of the NLRP3 inflammasome complex (Fig. 2C). We also tested the effect of MCC950, a well-known NLRP3 inhibitor, as a comparison. In contrast, even at a high concentration (10 μM), MCC950 did not show a substantial change in the BiFC signal, further suggesting that MCC950 is unable to disrupt preformed NLRP3 inflammasome complex (Fig. S1B). Additionally, an NLRP3 oligomerization cross-linking assay revealed that polydatin disrupted the formation of cage-NLRP3 (Fig. 2D). Furthermore, we observed that polydatin could inhibit the formation of the NLRP3 complex at 3 distinct stages, a capability not achievable with MCC950 (Fig. 2E to H). These results collectively indicate that polydatin exhibits a unique mechanism of action in inhibiting NLRP3 inflammasome formation.

Considering that polydatin comprises resveratrol and a glucoside moiety, we compared the effects of resveratrol and tapinarof (a resveratrol derivative) with those of polydatin on cage-NLRP3 formation. In contrast to polydatin, resveratrol demonstrated a weaker inhibitory effect on cage-NLRP3 formation, whereas tapinarof did not exhibit any noteworthy effects (Fig. S1C to E). These findings suggest that the glucoside moiety of polydatin may play a crucial role in its inhibitory effect on NLRP3 inflammasome formation.

As the formation of the NLRP3 double-ring cage is crucial for NLRP3 inflammasome activation, our investigation focused on determining whether polydatin influences NLRP3 inflammasome activation by assessing IL-1β secretion and CASP1 activation. Treatment of LPS-primed murine BMDMs or THP-1-derived macrophages with varying concentrations of polydatin led to a dose-dependent suppression of IL-1β release. Notably, this inhibition occurred without affecting IL-6 or tumor necrosis factor-α (TNF-α) levels, and without compromising cell viability (Figs. S2A to G and 3A to G). Although resveratrol and tapinarof demonstrated inhibitory effects on IL-1β secretion, their efficacy was notably lower than that of polydatin (Fig. S2I and J). These results further support the specificity of polydatin in targeting the NLRP3 inflammasome pathway.

Interestingly, polydatin did not hinder ATP- or carbonyl cyanide m-chlorophenylhydrazone (CCCP)-induced mitochondrial damage or reactive oxygen species production, both of which are antecedent signals in the activation of the NLRP3 inflammasome [21] (Fig. S3H to J). This observation suggests that polydatin likely acts directly on the NLRP3 complex, instead of interfering with antecedent signaling pathways.

Polydatin demonstrated dose-dependent inhibition of IL-1β and CASP1 cleavage without affecting the levels of pro-IL-1β or pro-CASP1 (Fig. S4A to C). CASP1 inactivation by polydatin was further confirmed by FAM-FLICA staining (Fig. S4D and E). Furthermore, importantly, polydatin did not influence AIM2 inflammasome activator poly(deoxyadenylic-deoxythymidylic) [poly(dA:dT)]-induced secretion of IL-1β or CASP1 activation (Figs. S2H and S5A and B).

In summary, polydatin treatment disrupts cage-NLRP3 formation and selectively inhibits IL-1β secretion mediated by canonical NLRP3 inflammasome activation.

### Polydatin inhibits the assembly of the NLRP3 inflammasome and promotes NLRP3 degradation

Next, our investigation aimed to determine whether polydatin could impede the formation of the ATP-induced endogenous NLRP3 inflammasome complex. Notably, polydatin significantly inhibited endogenous NLRP3-NEK7 and ASC-NLRP3 interactions (Fig. S4F and G). This observation was further validated in HEK293T cells, where interactions were detected between exogenously expressed HA-tagged NLRP3/GFP-tagged ASC and flag-tagged NLRP3 (Fig. S4H and I). Furthermore, polydatin treatment suppressed ASC oligomerization, a crucial step in ATP-induced NLRP3 activation [22], suggesting that polydatin interferes with steps preceding ASC oligomerization to hinder NLRP3 inflammasome activation (Fig. 2I).

We employed the blue native polyacrylamide gel electrophoresis to detect the effects of polydatin on NLRP3 oligomerization, which revealed a significant suppression by polydatin (Fig. 2J). Additionally, immunofluorescence (IF) analysis demonstrated that polydatin reduced the colocalization of ASC and CASP1, along with the ATP-induced formation of ASC specks [but not poly(dA:dT)], further indicating the inhibition of NLRP3 inflammasome assembly by polydatin treatment (Figs. S4J and 5C).

Upon examining the effect of polydatin on ASC, pro-CASP1, and NLRP3 expression under LPS+ATP/MSU/nigericin stimulation in BMDMs, we observed a dose-dependent decrease in NLRP3 protein levels, with no discernible effect on ASC, NEK7, or pro-CASP1 abundance (Fig. 2K and Fig. S6A and B). These findings suggest that polydatin specifically targets NLRP3 without affecting other components of the inflammasome. In light of these findings, we postulated that polydatin disrupts cage-NLRP3 and prevents NLRP3 inflammasome activation by down-regulating NLRP3. MCC950 is the most widely recognized small-molecule inhibitor of NLRP3. It selectively targets the “Walker B” motif within the NACHT domain of NLRP3, blocking ATP hydrolysis and consequently inhibiting NLRP3 inflammasome assembly and activation. Although some studies have shown that high concentrations of MCC950 can slightly reduce NLRP3 levels [23], we treated phorbol 12-myristate 13-acetate (PMA)-differentiated macrophages and HEK293T cells transfected with GFP-NLRP3 plasmid with 10 μM MCC950 and found that MCC950 did not affect NLRP3 levels (Fig. S6C and D). This observation highlights the unique mechanism of action of polydatin compared to MCC950.

It is interesting that polydatin selectively decreased NLRP3 expression without affecting NEK7 or ASC (Fig. S6E). To investigate whether polydatin reduced NLRP3 expression by blocking nuclear factor κB (NF-κB) activation, we examined the phosphorylation of NF-κB and the transcriptional levels of TNF-α, NLRP3, and IL-6. However, we observed no inhibitory effect of polydatin (Fig. S7A to G). These findings indicate that polydatin’s action on NLRP3 operates independently of NF-κB signaling. Therefore, we propose that polydatin may down-regulate NLRP3 by promoting NLRP3 degradation.

The suppression of NLRP3 expression by polydatin was diminished when BMDMs were treated with MG132 or chloroquine (CQ) (Fig. 2L). This observation was further confirmed by IF (Fig. S7H). To further determine whether the reduction in NLRP3-ASC and NLRP3-NEK7 interactions caused by polydatin is due to NLRP3 degradation, we treated BMDMs with CQ and MG132 and examined the endogenous NLRP3-NEK7 and NLRP3-ASC interactions. The results showed that polydatin could directly reduce the NLRP3-NEK7 and NLRP3-ASC interactions without down-regulating NLRP3, thereby inhibiting the assembly of the NLRP3 inflammasome (Fig. 2M and N). Collectively, polydatin significantly inhibits the activation of the NLRP3 inflammasome and specifically promotes NLRP3 degradation.

### Polydatin targets HSP90α for NLRP3 inflammasome inhibition

**Fig. 3.**
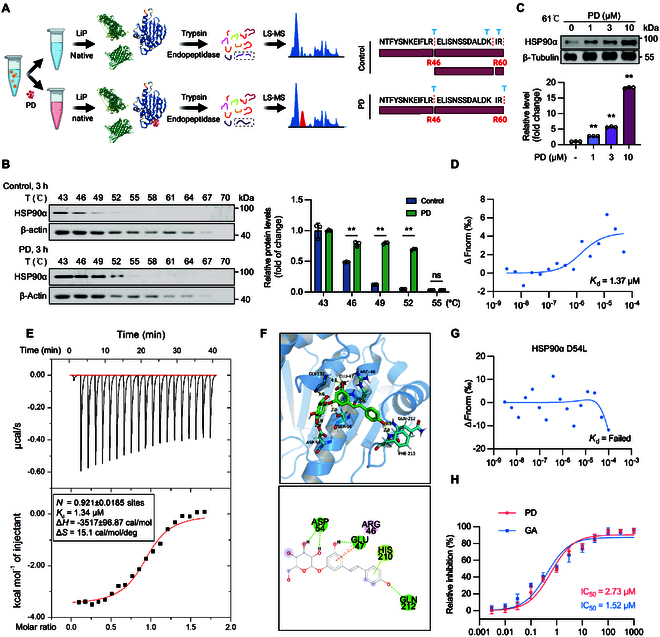
Polydatin directly binds to HSP90α. (A) Schematic representation of systematic identification of protein–small molecule interactions using Lip-SMap. (B) BMDMs were incubated with polydatin (DMSO as control) for 3 h, cells were collected, and CETSA was performed. (C) BMDMs were incubated with indicated concentrations of polydatin for 3 h, cells were collected, and CETSA was performed at 61 °C. (D) The affinity between polydatin and recombinant HSP90α protein was assessed by MST assay. (E) ITC enthalpogram of the interaction between polydatin and HSP90α at 25 °C. The titration curve shows the relationship between the molar ratio of HSP90α to the calculated concentration of polydatin in the assay. (F) Binding poses of polydatin against HSP90α (Protein Data Bank ID: 5H22). (G) The affinity between polydatin and recombinant HSP90α D54L mutant protein was assessed by MST assay. (H) Effect of polydatin and GA on the ATPase activity of HSP90α. After incubation of HSP90α with indicated different concentrations of polydatin or GA, ATPase was measured by Ultra-trace total ATPase test kit. Data are presented as mean ± SEM of 3 independent experiments.

Having confirmed that polydatin triggers NLRP3 degradation, we investigated the potential of polydatin to directly bind NLRP3, thereby suppressing NLRP3 inflammasome activation. To our surprise, the microscale thermophoresis (MST) assay revealed that there is no binding between polydatin and NLRP3 (Fig. S8A). To identify polydatin’s target, BMDM whole-protein lysates were treated with polydatin, followed by protein profiling analysis as described by Piazza et al. [24], which led to the identification of approximately 20 potential targets (Fig. 3A). We refined the list by evaluating the degree of enrichment in the mass spectrometry data and prioritizing proteins based on their known functions. In selecting our target, we focused on 2 key criteria—whether the protein could interact with NLRP3 inflammasome components and whether it played a role in maintaining complex protein assemblies. Among the proteins binding to polydatin, HSP90α, previously reported to interact with NLRP3 [25], was identified. The cellular thermal shift assay (CETSA) demonstrated that polydatin treatment increased the thermal stability of HSP90α with increasing temperature (Fig. 3B). Additionally, polydatin dose-dependently elevated the thermal stability of HSP90α at 61 °C (Fig. 3C), which was further corroborated in HSP90-overexpressing HEK293T cells (Fig. S8B and C).

To validate the direct binding of polydatin to HSP90α, peritoneal macrophages from polydatin-treated mice were subjected to CETSA, confirming the interaction between polydatin and HSP90α (Fig. S8D and E). Further validation was achieved via MST assay, which demonstrated a relatively higher binding affinity between polydatin and HSP90α, with a *K*_d_ (dissociation constant) of 1.37 μM for purified HSP90α and 5.8 μM for GFP-HSP90 lysate (Fig. 3D and Fig. S8F and G). Moreover, the isothermal titration calorimetry (ITC) assay confirmed that polydatin directly bound to HSP90α with a *K*_d_ of 1.34 μM (Fig. 3E). However, polydatin has no distinct effect on HSP90α levels (Fig. S8H to J). Furthermore, we sought to determine the specific binding site of polydatin on HSP90α. The molecular simulation analysis based on the crystal structure of the N-terminal domain of human HSP90α indicated a docking interaction between polydatin and the ATPase domain of HSP90α. The model suggests that a single molecule of polydatin binds to the N terminus of HSP90α, involving amino acid residues 46 to 212 (Fig. 3F). To further confirm the binding site of polydatin with HSP90α, we mutated the hydrophilic aspartic acid at position 54 of HSP90α to a hydrophobic leucine and found that polydatin could not bind to the HSP90α D54L mutant. This is likely due to the inability of polydatin to form a hydrogen bond with the hydrophobic leucine at position 54 in the HSP90α D54L mutant (Fig. 3G). Given the crucial role of HSP90α ATPase activity in inflammasome activation, the ATPase activity of HSP90α was examined. Polydatin inhibited HSP90α ATPase activity in a dose-dependent manner, with an approximate IC_50_ (median inhibitory concentration) of 2.73 μM, comparable to the activity of the classical HSP90α inhibitor geldanamycin (GA; IC_50_ = 1.52 μM) (Fig. 3H). The inhibitory effects of resveratrol and tapinarof on HSP90α ATPase activity were weaker than those of polydatin (Fig. S8K). This insight into the interactions between polydatin and its target indicates the crucial role of the glucoside moiety in mediating binding and inhibitory activity.

To further confirm that polydatin targets HSP90α to inhibit NLRP3 inflammasome activation, we utilized HSP90α KO BMDMs and found that polydatin could not further inhibit IL-1β secretion in HSP90α KO BMDMs (Fig. S9A to D), thereby confirming that HSP90α is the direct target protein of polydatin. Given that HSP90β, another isoform of HSP90, is highly homologous to HSP90α, we investigated whether polydatin binds to HSP90β via MST assay. The results showed that polydatin specifically binds to HSP90α, but not to HSP90β (Fig. S9E). To further investigate this distinction, we compared the amino acid sequences of HSP90α and HSP90β and identified significant differences near the predicted binding site for polydatin. Through molecular simulation analysis based on the crystal structure of the N-terminal domain of human HSP90β, we found that the conformation of HSP90β hinders its effective binding to polydatin (Fig. S9F). In summary, these analyses demonstrate that polydatin directly interacts with HSP90α at both cellular and molecular levels.

### Polydatin disrupts the integrity of 3 forms of the NLRP3 inflammasome complex

**Fig. 4.**
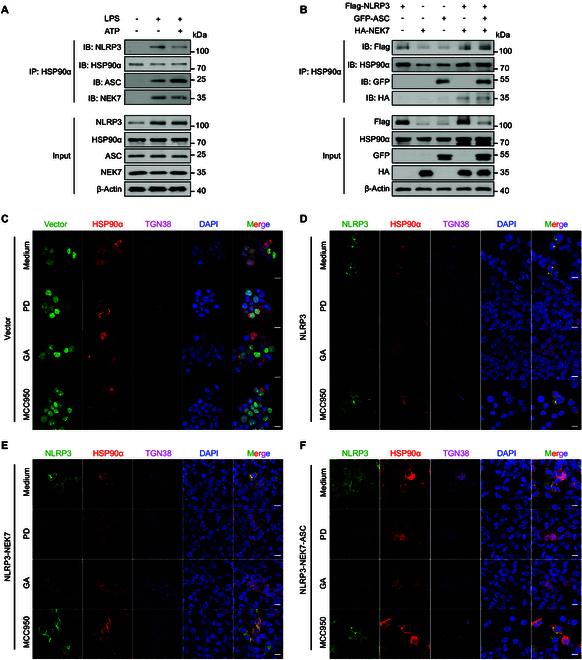
HSP90α stabilized the formation of NLRP3 complexes in 3 stages. (A) An endogenous IP with HSP90α antibody was performed in LPS and/or ATP-stimulated BMDMs. (B) HEK293T cells were transfected with HA-NEK7, flag-NLRP3, or GFP-ASC alone or cotransfected with HA-NEK7 and flag-NLRP3, or HA-NEK7, flag-NLRP3, and GFP-ASC. IP and Western blotting analysis of the interaction between HSP90α and 3 stages of NLRP3 complexes. (C) GFP-Vector, (D) Cage-NLRP3, (E) NLRP3-NEK7 complex, and (F) NLRP3-NEK7-ASC complex were reproduced in HEK293T cells. Colocalization of HSP90α and 3 stages of NLRP3 complexes was determined by IF. Scale bar, 10 μm.

As a highly abundant and evolutionarily conserved molecular chaperon, HSP90α mainly serves as a cofactor in folding “polyproteins” into stable structures [26,27]. This chaperone plays a crucial role in maintaining the stability and functionality of various cellular proteins. To explore whether HSP90α promotes the stability of cage-NLRP3, NLRP3-NEK7, and NLRP3-NEK7-ASC complexes, we examined the interactions of HSP90α with these complexes. In resting BMDMs, HSP90α did not bind to NEK7. However, upon LPS stimulation, NLRP3 undergoes oligomerization, with NEK7 forming connections between the LRR domains of adjacent NLRP3 proteins [15]. Subsequent coimmunoprecipitation revealed an association between HSP90α and NEK7. Similarly, HSP90α did not interact with ASC under physiological conditions; however, costimulation with LPS and ATP led to the coimmunoprecipitation of ASC with HSP90α, indicating that HSP90α stabilized both NLRP3-NEK7 and NLRP3-ASC-NEK7 complexes (Fig. 4A). This observation was validated in HEK293T cells (Fig. 4B), establishing that HSP90α engages with cage-NLRP3, the NLRP3-NEK7 complex, and the NLRP3-ASC-NEK7 complex.

Next, we investigated whether polydatin weakens the interaction between HSP90α and these 3 NLRP3 complexes, thereby disrupting the stable structure of the NLRP3 complexes. We employed IF microscopy to visualize the colocalization and structural integrity of these complexes. The results demonstrated that HSP90α indeed colocalized with the cage-NLRP3, NLRP3-NEK7, and NLRP3-NEK7-ASC complexes, with a clear formation of NLRP3 complexes observed in the cytoplasm. Both GA, a recognized HSP90 inhibitor, and polydatin effectively hindered the interaction between HSP90α and the cage-NLRP3, NLRP3-NEK7, and NLRP3-ASC-NEK7 complexes, leading to the disappearance of these NLRP3 complex structures. In contrast, MCC950 appeared to stabilize NLRP3 in a cage-like structure in a self-restricting manner, but failed to disrupt the 3 states of NLRP3 complexes (Fig. 4C to F).

These findings suggest that compared to other NLRP3 inhibitors, polydatin disrupts the integrity of 3 forms of the NLRP3 inflammasome complex.

### HSP90α inhibition by polydatin disrupts the integrity of NLRP3 inflammasome complex

**Fig. 5.**
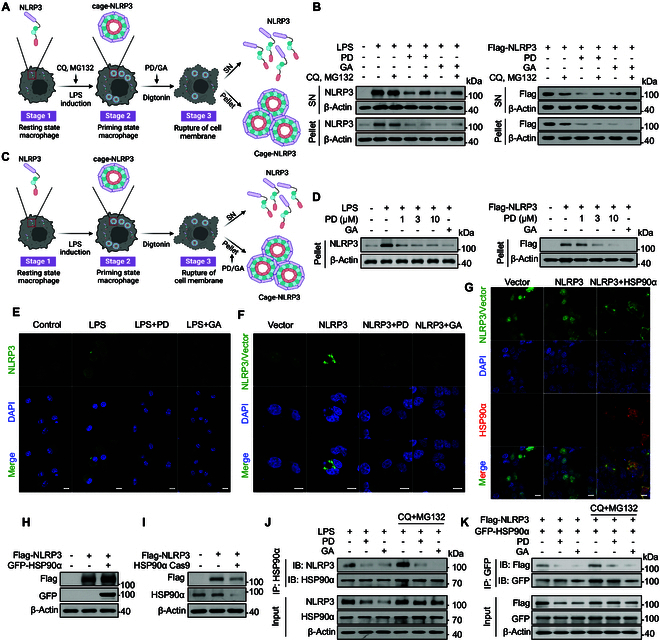
HSP90α inhibition directly disrupts cage-NLRP3. (A) BMDMs were treated with 20 μM MG132 and 30 μM CQ for 2 h, and then 100 ng/ml LPS for 3 h followed by 10 μM polydatin or 10 μM GA treatment for 1 h. HEK293T cells transfected with flag-tagged NLRP3 or BMDMs were treated with 20 μM MG132 and 30 μM CQ for 2 h, followed by 10 μM polydatin or 10 μM GA treatment for 1 h. The cells were then treated with 40 μg/ml digitonin on ice to permeabilize the plasma membrane. (B) The NLRP3 monomers and double-ring cage-NLRP3 oligomers were separated and examined by immunoblot. (C) BMDMs were stimulated with 100 ng/ml LPS for 3 h (above). HEK293T cells were transfected with flag-NLRP3 (below). The cells were treated with 40 μg/ml digitonin on ice to permeabilize the plasma membrane. (D) The pellets were treated with 10 μM polydatin or 10 μM GA for 1 h, and the NLRP3 expression was analyzed by immunoblot. (E) BMDMs stimulated with 100 ng/ml LPS were treated with 10 μM polydatin or 10 μM GA treatment for 1 h. NLRP3 expression was examined using IF. (F) HEK293T cells transfected with GFP-NLRP3 were treated with 10 μM polydatin or 10 μM GA. GFP expression was observed by confocal microscopy. (G) The stable HSP90α KO HEK293T cell line was conducted by CRISPR-Cas9. HSP90α KO HEK293T cells were transfected with GFP-NLRP3 and mCherry-HSP90α and then observed by confocal microscopy. (H and I) HEK293T cells transfected with flag-NLRP3 were cotransfected with GFP-HSP90α or infected with HSP90α Cas9 lentivirus. The expression of HSP90α and NLRP3 was determined by immunoblot. (J) An endogenous IP with HSP90α antibody was performed in LPS-primed BMDMs treated with or without CQ and MG132 in the presence of polydatin and GA. Western blot analysis of HSP90α and NLRP3. (K) HEK293T cells were transfected with flag-NLRP3 and GFP-HSP90α and treated with or without CQ and MG132. Interaction between HSP90α and NLRP3 in the presence of polydatin and GA was examined by IP. Data are representative of 3 independent experiments. Scale bar, 10 μm.

As previously demonstrated, the introduction of full-length NLRP3 into HEK293T cells lacking ASC or pro-CASP1 results in the spontaneous formation of inactive, membrane-localized double-ring cage-like structures of NLRP3. However, in BMDMs, NLRP3 expression increased rapidly upon LPS stimulation and not all NLRP3 formed double-ring cage-like structures. Under physiological conditions, both NLRP3 monomers and double-ring cage-NLRP3 coexist in cells. Therefore, we investigated whether polydatin primarily targeted monomeric NLRP3 or cage-NLRP3 structures. After digitonin permeabilization following polydatin treatment, NLRP3 monomers and double-ring cage-NLRP3 oligomers were separated, resulting in the release of NLRP3 monomers into the supernatant, whereas the cage-NLRP3 oligomers remained in the pellet (Fig. 5A). Polydatin reduced the levels of both NLRP3 monomers and cage-NLRP3 oligomers, and treatment with CQ and MG132 counteracted the pro-degradation effect of polydatin on NLRP3 monomers, but did not reverse the disruption of cage-NLRP3. These findings suggested that polydatin treatment directly facilitated the degradation of NLRP3 monomers and destabilized cage-NLRP3, transforming cage-NLRP3 into NLRP3 monomers (Fig. 5B).

Next, we separated the pellets obtained after digitonin permeabilization (maintaining the basic cellular structure) and treated them with polydatin to assess whether polydatin could attenuate the stability of NLRP3 in a cage structure (Fig. 5C). As expected, the level of cage-NLRP3 decreased following polydatin treatment, indicating that polydatin directly dismantled the cage-NLRP3 oligomers (Fig. 5D). To further validate these findings, we employed IF analysis in both BMDMs and HEK293T cells, which corroborated our biochemical results (Fig. 5E and F).

Subsequently, we investigated the mechanism by which polydatin-induced inhibition of HSP90α affected the stability of NLRP3. HSP90α played a crucial role in up-regulating the expression of NLRP3, and its absence reduced the levels of NLRP3 (Fig. 5G to I). Our findings suggest that inhibition of HSP90α disrupts protein–protein interactions with co-chaperones and client substrates. Furthermore, pharmacological inhibition of HSP90α leads to the dissociation of HSP90α and NLRP3, where the association of HSP90α and NLRP3 is indispensable for inflammasome activation. Therefore, we treated BMDMs with or without polydatin before LPS stimulation to determine whether polydatin could attenuate the stabilizing effect of HSP90α on NLRP3. Our findings indicate that polydatin disrupts the interaction between NLRP3 and HSP90α, leading to a reduction in NLRP3 levels (Fig. 5I).

To validate whether the reduced interaction between HSP90α and NLRP3 was mainly attributed to decreased NLRP3 expression, we employed CQ and MG132 to inhibit NLRP3 degradation in BMDMs and yielded consistent outcomes (Fig. 5I). To further corroborate our findings, we examined the effect of polydatin in HEK293T cells and detected an interaction between exogenously expressed GFP-HSP90α and Flag-tagged NLRP3 (Fig. 5J). Our results showed that polydatin similarly reduced the stabilizing effect of HSP90α on NLRP3 in this system. In summary, the suppressive action of polydatin on HSP90α disrupts the interaction between HSP90α and NLRP3, consequently attenuating the stabilizing effect of HSP90α on NLRP3, reducing the level of NLRP3 monomer, and directly dismantling preformed cage-NLRP3 complexes. These findings provide novel insights into the molecular mechanisms underlying polydatin’s anti-inflammatory effects and suggest potential therapeutic strategies for targeting the HSP90α-NLRP3 interaction in inflammatory disorders.

### HSP90α inhibition facilitates Cbl-b-mediated ubiquitination of NLRP3

**Fig. 6.**
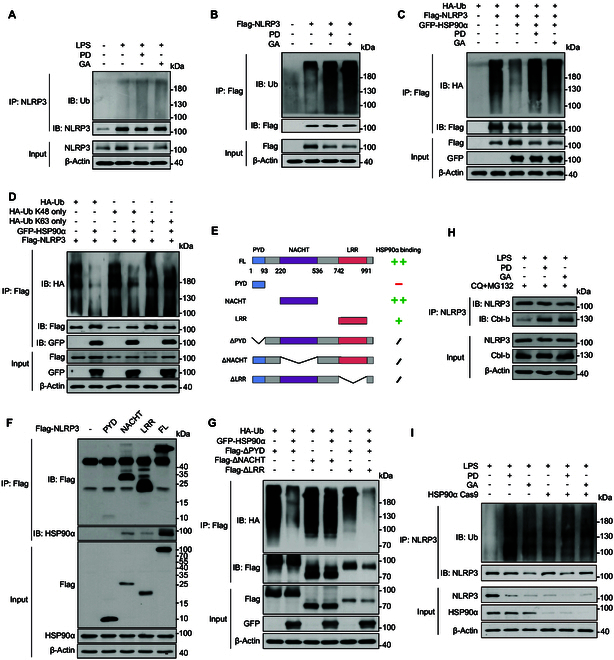
HSP90α inhibition promoted the ubiquitination of NLRP3 mediated by Cbl-b. (A) BMDMs treated with 10 μM polydatin or GA were stimulated with 100 ng/ml LPS for 3 h, or (B) HEK293T cells transfected with flag-NLRP3 were treated with 10 μM polydatin or GA. NLRP3 ubiquitination level was analyzed by immunoblot. (C) HEK293T cells were transfected with various combinations (above lanes) of plasmids encoding flag-NLRP3, GFP-HSP90α, and HA-Ub, and immunoblot analysis of NLRP3 ubiquitination (detected by anti-HA antibody) in cell lysates immunoprecipitated with anti-flag agarose. (D) HEK293T cells were transfected with various combinations (above lanes) of plasmids encoding flag-NLRP3, GFP-HSP90α and HA-Ub, HA-Ub K48 only, or K63 only. Immunoblot analysis of NLRP3 ubiquitination (detected by anti-HA antibody) in cell lysates immunoprecipitated with anti-flag agarose. (E) Schematic representation of NLRP3 WT and deletion mutants. (F) HEK293T cells were transfected with various combinations (above lanes) of plasmids encoding NLRP3 truncations as indicated. Immunoblot analysis of interaction between HSP90α and NLRP3 truncations in lysates immunoprecipitated with anti-flag agarose. (G) HEK293T cells were transfected with various combinations (above lanes) of plasmids encoding HA-Ub, GFP-HSP90α, and NLRP3 truncations as indicated. Immunoblot analysis of ubiquitination of NLRP3 truncations (detected by anti-HA antibody) in cell lysates immunoprecipitated with anti-flag agarose. (H) BMDMs treated with CQ and MG132 were added 10 μM polydatin or GA and then exposed to 100 ng/ml LPS for 3 h. An endogenous IP with NLRP3 antibody was performed. (I) BMDMs infected with HSP90α Cas9 lentivirus were treated with 10 μM polydatin or GA, followed by 100 ng/ml LPS for 3 h. NLRP3 ubiquitination level was analyzed by immunoblot. Data are representative of 3 independent experiments.

Considering that deubiquitination of NLRP3 facilitates NLRP3 inflammasome activation [20,28], we investigated whether the inhibition of HSP90α by polydatin influenced NLRP3 ubiquitination. Our results revealed that polydatin significantly induced NLRP3 ubiquitination in both BMDMs and HEK293T cells (Fig. 6A and B). HSP90α overexpression notably reduced NLRP3 ubiquitination, whereas polydatin-induced HSP90α inhibition restored NLRP3 ubiquitination (Fig. 6C). Further examination of the polyubiquitination pattern of NLRP3 regulated by HSP90α showed that HSP90α overexpression specifically decreased the K48-linked polyubiquitination of NLRP3, similar to the reduction in wild-type (WT) ubiquitin-linked polyubiquitination, without affecting K63-linked polyubiquitination (Fig. 6D).

To understand the mechanisms by which HSP90α influences NLRP3 ubiquitination, we identified the domains through which HSP90α binds to NLRP3 (Fig. 6E). As depicted in Fig. 6F, HSP90α interacted with the NLRP3 NACHT and LRR domains and mitigated the ubiquitination of the NLRP3 NACHT domain (Fig. 6G).

Previous studies have suggested that Cbl-b interacts with the NACHT domain of NLRP3 and facilitates its K48-linked ubiquitination [29], promoting its degradation. Therefore, we investigated whether HSP90α competitively binds to NLRP3 with Cbl-b, thus shielding NLRP3 from ubiquitination. Our experiments showed that following polydatin treatment, the interaction between NLRP3 and Cbl-b was significantly intensified (Fig. 6H). To further confirm the role of HSP90α in this process, we employed CRISPR-Cas9 technology to generate HSP90α KO cells and assessed the impact of polydatin on NLRP3 ubiquitination in these cells The results revealed a significant enhancement of NLRP3 ubiquitination upon polydatin treatment in WT cells. However, in HSP90α KO cells, the promoting effect of polydatin on NLRP3 ubiquitination was nearly abolished (Fig. 6I). These results demonstrate that HSP90α inhibition caused by polydatin undermines the stabilizing influence of HSP90α on NLRP3, augmenting Cbl-b-mediated K48-linked poly-ubiquitination of NLRP3, thereby promoting NLRP3 degradation. This finding reveals a novel regulatory mechanism of NLRP3 inflammasome activation and highlights potential therapeutic strategies for inflammasome-related diseases.

### Polydatin challenges the stability of the NLRP3 CAPS-associated mutants by inhibiting HSP90α

**Fig. 7.**
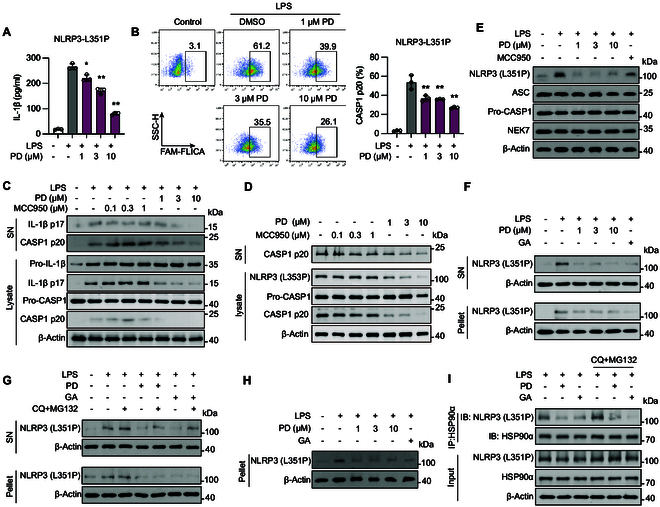
HSP90α inhibition caused by polydatin posed a challenge to the stability of the NLRP3 CAPS-associated mutants. (A to H) NLRP3^lyz−/−^ BMDMs were recombined with mouse NLRP3 L351P mutant (L351P-BMDM). (A) L351P-BMDMs were treated with indicated concentrations of polydatin followed by 100 ng/ml LPS for 3 h. IL-1β in the supernatant was determined by ELISA. (B) CASP1 activation was determined by FAM-FLICA staining. (C) Cleaved IL-1β (p17), activated CASP1 (p20) in culture supernatants (SN), and pro-IL-1β, IL-1β p17, pro-CASP1, and CASP1 p20 in lysates of NLRP3 L351P BMDMs were analyzed by immunoblot. (D) HEK293T cells were cotransfected with human NLRP3 (L353P), ASC, NEK7, and pro-CASP1 to reconstruct the human inflammasome system. CASP1 (p20) in culture supernatants and CASP1 (p20), pro-CASP1, and NLRP3 (L353P) in lysates were analyzed by immunoblot. (E) Western blot analysis of NLRP3 (L351P), ASC, NEK7, and pro-CASP1. (F) L351P-BMDMs were treated with indicated concentrations of polydatin or 10 μM GA for 1 h, followed by 100 ng/ml LPS for 3 h. The cells were then treated with 40 μg/ml digitonin on ice to permeabilize the plasma membrane. The NLRP3 (L351P) monomers and double-ring cage-NLRP3 oligomers were separated and examined by immunoblot. (G) L351P-BMDMs were treated with 20 μM MG132 and 30 μM CQ for 2 h, and then 10 μM polydatin or 10 μM GA treatment for 1 h followed by 100 ng/ml LPS for 3 h. The NLRP3 (L351P) monomers and double-ring cage-NLRP3 oligomers were separated and examined by immunoblot. (H) L351P-BMDMs were stimulated by 100 ng/ml LPS for 3 h. The cells were treated with 40 μg/ml digitonin on ice to permeabilize the plasma membrane. The pellets were treated with 10 μM polydatin or 10 μM GA for 1 h, and the NLRP3 (L351P) expression was analyzed by immunoblot. (I) An endogenous IP with HSP90α antibody was performed in LPS-primed BMDMs treated with or without CQ and MG132 in the presence of polydatin and GA. Western blot analysis of HSP90α and NLRP3 (L351P). Data are presented as mean ± SEM of 3 independent experiments. *P < 0.05, **P < 0.01 versus LPS.

As mentioned above, MCC950 failed to mitigate the activity of the CAPS-associated NLRP3 mutant L351P (corresponding to the human L353P mutant). To address this limitation, we explored the potential of polydatin in preventing NLRP3 inflammasome activation caused by gain-of-function mutations. Surprisingly, polydatin effectively mitigated the activity of the CAPS-associated NLRP3 mutant L351P, as evidenced by the inhibition of IL-1β secretion and CASP1 maturation (Fig. 7A to C). Furthermore, we constructed a plasmid carrying the human NLRP3 L353P mutation and reconstituted the inflammasome system (NLRP3 L353P) in HEK293T cells. We found that polydatin effectively inhibits the activation of CASP1 and the production of CASP1 p20 induced by NLRP3 L353P activation (Fig. 7D).

To further elucidate the mechanism underlying polydatin’s effect on CAPS-associated NLRP3 mutants, treatment with polydatin resulted in a dose-dependent decrease in the abundance of the NLRP3 L351P mutant (Fig. 7E). Additionally, polydatin hindered the interaction between the NLRP3 L351P mutant and HSP90α, inhibiting the stabilizing effect of HSP90α. This disruption of the interaction facilitated the degradation of NLRP3 L351P mutant monomers and destabilized cage-NLRP3, akin to its effect on WT NLRP3 (Fig. 7F to I). Our findings demonstrate that polydatin exhibits comparable inhibitory effects on CAPS-associated NLRP3 mutants as on WT NLRP3, underscoring its efficacy. This suggests that polydatin surpasses MCC950 in controlling aberrant NLRP3 activation, positioning it as a promising candidate for treating disorders linked to gain-of-function mutations in NLRP3. In summary, our study reveals a novel mechanism by which polydatin targets both WT and mutant NLRP3 through HSP90α inhibition, offering a potential therapeutic approach for CAPS and other NLRP3-related disorders.

### HSP90α inhibition alleviates NLRP3-related inflammatory responses in vivo

AP is a severe disease with a high mortality rate, and the NLRP3 inflammasome plays a crucial role in its pathogenesis [30]. To further confirm the role of NLRP3 inflammasome in AP pathogenesis, we established an AP model using both WT and macrophage-specific NLRP3-KO (NLRP3^lyz−/−^) mice. We found that NLRP3^lyz−/−^ mice exhibited milder symptoms of AP compared to WT mice, reflected by a significantly reduced increase in serum lipase and amylase levels (Fig. S10A and B). As an indicator of systemic inflammation, the myeloperoxidase (MPO) activity in the pancreas and lung tissues was lower in NLRP3^lyz−/−^ AP model mice (Fig. S10C). Additionally, NLRP3^lyz−/−^ mice exhibited a lower proportion of CD4^+^CD69^+^ T cells in the spleen compared to WT mice, correlating with reduced tissue damage in the lungs and pancreas (Fig. S10D and E).

**Fig. 8.**
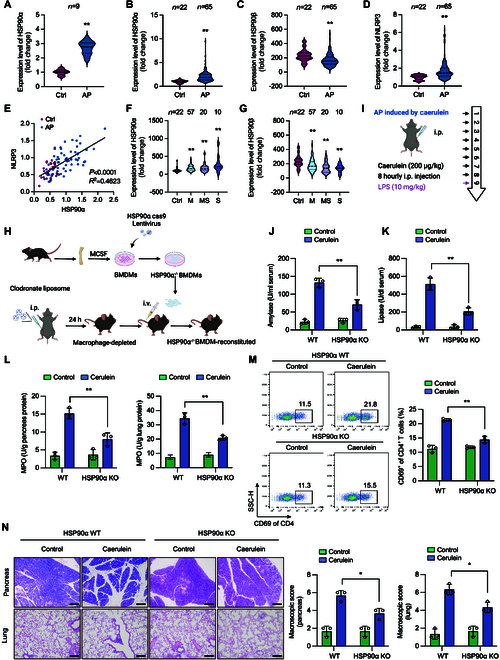
HSP90α plays a critical role in the pathological process of AP. (A) GEO (Gene Expression Omnibus) database (GSE3644) of HSP90α expression profiles between cerulein-induced mice AP models and healthy control. (B to D) GEO database (GSE194331) of (B) HSP90α, (C) HSP90β, and (D) NLRP3 expression profiles between AP patients and healthy control. (E) Correlation between HSP90α and NLRP3 expression level in AP patients and healthy control. M, mild AP; MS, moderately severe AP; S, severe AP. (F and G) GEO database (GSE194331) of (F) HSP90α and (G) HSP90β expression profiles among patients with varying degrees of AP and healthy controls. (H) Schematic of the depletion and reconstitution of mouse macrophages. (I to N) AP was induced by cerulein stimulation in mice. (I) Schematic diagram illustrating the establishment of mouse AP model. (J) Serum amylase and (K) lipase activities were determined to reflect pancreatic damage and disease severity. (L) MPO level in lungs and pancreases was analyzed to reflect the severity of systemic inflammation. (M) Representative flow cytometry plots of CD4^+^ CD69^+^ T cells in spleen. (N) Representative hematoxylin and eosin (H&E) staining of mouse pancreas and lung tissues. Scale bar, 100 μm. Data are presented as mean ± SEM of 3 mice in each group. *P < 0.05, **P < 0.01 versus as indicated.

To evaluate the potential involvement of HSP90α in AP, we analyzed the expression levels of HSP90α in human peripheral blood and mouse pancreatic tissue using publicly available RNA-sequencing data [31,32]. The data showed that the levels of HSP90α in the pancreas of AP mice were significantly higher than in healthy mice (Fig. 8A). Similarly, in human peripheral blood samples, HSP90α expression in AP patients was significantly higher than in healthy controls, with corresponding significant increases in NLRP3. In contrast, HSP90β showed an opposite trend (Fig. 8B to D). Furthermore, HSP90α expression was positively correlated with NLRP3 (Fig. 8E). When stratifying AP patients into mild AP, moderate-severe AP, and severe AP groups, we found that HSP90α levels progressively increased with the severity of AP (Fig. 8F). HSP90β expression gradually decreased with the increasing severity of AP, further supporting our observations (Fig. 8G). These findings collectively demonstrate a potential role of HSP90α in the pathogenesis of AP, possibly through its interaction with the NLRP3 inflammasome, suggesting its potential as a novel therapeutic agent for AP treatment. Due to the infertility observed in HSP90α KO mice, we employed clodronate liposomes to deplete macrophages in mice, and subsequently reintroduced HSP90α KO macrophages into the mice via intravenous injection through the tail vein, thereby mimicking the HSP90α KO phenotype as closely as possible (Fig. 8H). The macrophage depletion and reconstitution approach was validated by flow cytometry analysis. Similarly, mice receiving HSP90α KO BMDMs exhibited milder AP, showing the same characteristics as NLRP3^lyz−/−^ mice: a weaker increase in serum amylase and lipase levels, along with less damage to the pancreas and lung tissues (Fig. 8J to N). These findings suggest that HSP90α plays a crucial role in NLRP3 inflammasome-related diseases such as AP. More specifically, our data indicate that HSP90α functions through macrophage-specific regulation of NLRP3 inflammasome activation.

**Fig. 9.**
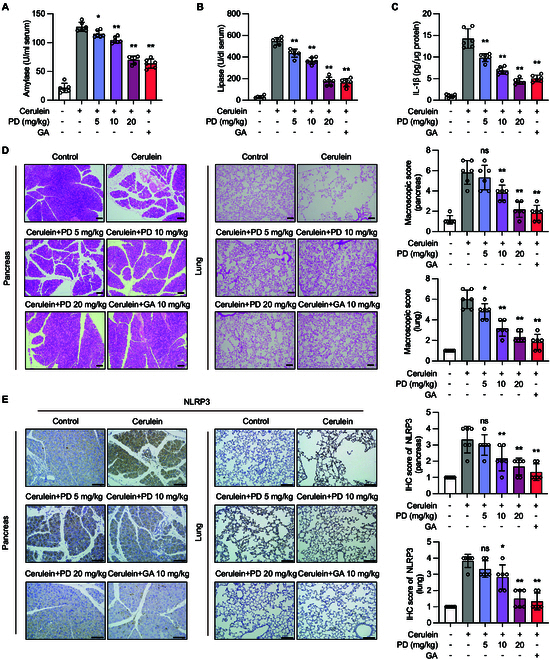
Polydatin ameliorates cerulein-induced AP in mice. (A to E) AP was induced by cerulein stimulation in mice. Mice were treated with indicated concentrations of polydatin (intragastric) or 10 mg/kg GA before cerulein stimulation. (A) Serum amylase and (B) lipase activities were determined to reflect pancreatic damage and disease severity. (C) The level of IL-1β in pancreas tissues was determined by ELISA. (D) Representative H&E staining of mouse pancreas and lung tissues. (E) Representative image of mouse pancreas and lung tissue sections for NLRP3 staining. Scale bar, 100 μm. Data are presented as mean ± SEM of 6 mice in each group. *P < 0.05, **P < 0.01 versus as indicated.

To validate the in vivo efficacy of polydatin, we used a cerulein-induced AP model to assess its therapeutic impact on the development of NLRP3 inflammasome-associated diseases. We administered cerulein (50 μg/kg) intraperitoneally at hourly intervals for a total of 7 injections to establish the AP model [33]. Polydatin caused a significant reduction in serum amylase and lipase activities, comparable to the effect of GA (Fig. 9A and B). Moreover, polydatin administration lowered the levels of TNF-α in the serum and IL-1β in pancreatic tissues compared to those in the model group (Fig. 9C and Fig. S11A). Additionally, the MPO activity in both pancreatic and lung tissues decreased following polydatin treatment (Fig. S11B). The total number of CD4^+^CD69^+^ T cells in the spleen also decreased after polydatin treatment (Fig. S11C). Paraffin-embedded pancreatic and lung tissue sections were used to assess the effect of polydatin on pancreatic and lung injury. Polydatin treatment attenuated intra-pancreatic necrosis and lung injury (Fig. 9D and E and Fig. S11D). Importantly, polydatin reduced the level of inflammatory cytokines and infiltration of inflammatory cells in pancreatic and lung tissues (Fig. S12). Therefore, polydatin treatment effectively attenuated cerulein-induced AP in mice by suppressing the formation of the NLRP3 inflammasome. These findings collectively demonstrate the therapeutic potential of polydatin in treating AP through its inhibitory effects on the NLRP3 inflammasome, highlighting its promise as a novel treatment strategy for this severe condition.

To further elucidate the mechanism of action, we assessed the therapeutic effect of polydatin on peritoneal inflammation in HSP90α KO mice by establishing a mouse model of peritoneal inflammation through intraperitoneal injection of MSU. Our findings revealed that HSP90α KO resulted in a notable reduction in peritoneal inflammation, marked by diminished peritoneal exudate cell (PEC) infiltration and lower IL-1β levels in the peritoneal cavity, indicating a potential suppression of NLRP3 inflammasome activation. Furthermore, our data demonstrated that polydatin treatment did not elicit further attenuation in peritoneal inflammation in HSP90α KO mice, implying a shared target for polydatin and HSP90α KO (Fig. S13). Collectively, these results further corroborate that polydatin inhibits NLRP3 inflammasome activation in vivo by suppressing HSP90α.

## Discussion

The NLRP3 inflammasome, as one of the most extensively studied member of the inflammasome family, is closely related to the occurrence and development of nearly all inflammation-related diseases [34]. Its activation is a complex process involving multiple steps and regulatory mechanisms. This process can be broadly divided into several key stages: Activation of the NLRP3 inflammasome involves monomer NLRP3 transcription, formation of inactive cage-like structures (cage-NLRP3), and subsequent transition to active NLRP3 inflammasome discs [16]. After deubiquitylation, monomer NLRP3 forms cage-NLRP3. Once bound to dTGN, cage-NLRP3 undergoes conformational changes and forms the NLRP3-NEK7 complex and, subsequently, the NLRP3-NEK7-ASC complex. This stepwise assembly process is critical for the proper activation of the inflammasome. MCC950 reduces the ATPase activity of NLRP3 by interacting with the cleft between the NBD, HD1, WHD, HD2, and trLRR subdomains of NLRP3. Specifically, the interaction of MCC950 markedly stabilizes the relative position between the NACHT and trLRR domains [35]. MCC950 fills the fissure where its central sulfonamide group engages with the Walker B motif of NACHT and is wedged between 2 arginine residues (hArg351 and hArg578). The engagement of MCC950 with residues from 5 distinct subdomains in this fissure and the stabilization of their conformational alignment led to its conformational locking, which impaired its ability to undergo conformational changes, resulting in the inactivation of the NLRP3 inflammasome. However, the formation of a cage-like decamer structure by NLRP3 creates spatial constraints that prevent MCC950 from penetrating and binding effectively, indicating that MCC950 cannot exert sufficient inhibition on the assembled NLRP3 complex [17,18]. Furthermore, severe nephrotoxicity and poor anti-inflammatory efficacy observed in clinical trials ultimately led to the termination of MCC950’s development, raising serious concerns about the feasibility of NLRP3 inhibitor development. This underscores the urgent need for alternative strategies to target NLRP3 after its assembly.

*Polygonum cuspidatum*, a herb commonly utilized in traditional Chinese medicine, has been widely used for the management of inflammatory diseases [36*–*38]. Polydatin, a resveratrol glucoside isolated from the roots of *P. cuspidatum* Sieb. et Zucc [39], has been reported to effectively alleviate fructose-induced liver inflammation. In this study, we established a screening system to identify compounds targeting the cage-NLRP3 complex and identified polydatin as a potent inhibitor of cage-NLRP3 formation. Notably, polydatin displayed efficacy against both WT and autoactivated NLRP3, including CAPS-associated mutants such as L351P, which MCC950 struggled to efficiently suppress [40]. More importantly, this broader inhibitory spectrum of polydatin represents a significant therapeutic advantage over existing NLRP3 inhibitors in treating various inflammatory conditions. Furthermore, polydatin has demonstrated therapeutic benefits in mouse models of NLRP3 inflammasome-associated diseases, specifically cerulein-induced AP. To circumvent the limitations posed by the infertility of HSP90α KO mice, we employed clodronate liposomes to deplete macrophages in mice, and subsequently reintroduced HSP90α KO macrophages into the mice via intravenous injection through the tail vein, aiming to simulate HSP90α KO mice to the fullest extent possible. This innovative approach allowed us to overcome the limitations of traditional KO models and study the specific role of HSP90α in macrophages. Using this established model system, we further confirmed through constructing a mouse peritonitis model that polydatin inhibits NLRP3 inflammasome activation in vivo by suppressing HSP90α. Our investigations revealed that polydatin targeted HSP90α, disrupting its chaperone function and weakening its stabilizing effect on the 3 forms of NLRP3 complexes. This disruption leads to the structural breakdown of these NLRP3 complexes and enhances Cbl-b-mediated K48-linked polyubiquitination of NLRP3, ultimately resulting in its degradation. This mechanism of action achieves comprehensive inhibition at different stages of NLRP3 inflammasome activation, making it a promising candidate for the management of AP, peritonitis, and other inflammasome-associated diseases [41].

Based on our findings and the analysis of the complex structure at different stages of NLRP3 activation by Wu et al., we found that HSP90α is the driving force behind the formation of the cage-NLRP3, NLRP3-NEK7, and NLRP3-NEK7-ASC complexes [16]. Furthermore, HSP90α maintains the stability of the NLRP3-ASC-CASP1 complex, which is involved in inflammation when the NLRP3 inflammasome is fully assembled and activated. Although the structure of the NLRP3-ASC-CASP1 complex has not yet been fully elucidated, our current findings strongly suggest that HSP90α acts as a “safe guardian”, facilitating the formation of the NLRP3-ASC-CASP1 complex, and maintains its structure during the inflammatory process to promote the progression of inflammation.

HSP90 comprises 2 isoforms, namely, HSP90α (inducible form/major form) and HSP90β (constitutive form/minor form), both of which serve as molecular chaperones for various client proteins [27,42–45]. These proteins often require HSP90 to achieve a proper conformation and functional state. HSP90 plays a crucial role in the folding and conformational regulation of various clinically relevant signal transduction molecules [46]. The essential nature of HSP90 in protein homeostasis makes it an attractive therapeutic target. Recent studies have indicated that disrupting HSP90β function may provide a possibility to specifically target NLRP3 disease activity without affecting the WT NLRP3 response. However, cells lacking HSP90β retain the ability to secrete IL-1β, and there is an up-regulation of intracellular HSP90α expression, which indicates a compensatory mechanism involving HSP90α, suggesting a certain role of HSP90α in the activation of NLRP3 inflammasomes [47]. It is important to note that upon stimulation of macrophages, the NLRP3 inflammasome assembly and activation occur when the cells are under stress conditions. Here, HSP90α, but not HSP90β, was inhibited by polydatin, resulting in the degradation of NLRP3 monomers and inhibition of cage-NLRP3 formation. Even after inhibiting the degradation of NLRP3 monomers by MG132, polydatin continued to reduce the formation of cage-like NLRP3, suggesting that HSP90α functions as a chaperone to stabilize both NLRP3 monomers and oligomers and contribute to NLRP3 inflammasome activation. The distinct roles of these highly homologous isoforms in inflammasome regulation remain to be fully elucidated. This uncertainty highlights the complexity of HSP90 isoform-specific functions in inflammasome regulation and underscores the need for more detailed mechanistic studies. Theoretically, pharmacologic targeting of HSP90α could potentially play a role in destabilizing monomeric NLRP3, cage-NLRP3, and disc NLRP3, thereby facilitating intervention at various stages of the inflammatory process. This multi-level intervention strategy could provide more effective therapeutic outcomes. Several HSP90 inhibitors have been developed and evaluated in both preclinical and clinical studies [48,49]. These inhibitors target HSP90 and disrupt its chaperone function to inhibit the growth and survival of cancer cells, which rely on HSP90 to stabilize key signaling proteins. Among various HSP90 inhibitors, 17-allylamino-17-demethoxygeldanamycin (17-AAG), an analog of GA, represents one of the most well-known HSP90 inhibitors, with promising results in preclinical studies [50]. However, its clinical development faces challenges due to formulation issues and toxicity concerns [51]. Other HSP90 inhibitors in the development pipeline include ganetespib [52], luminespib [53], onalespib [54], and AUY922 [55]. Although these inhibitors have shown varying degrees of efficacy in preclinical studies and early phase clinical trials against different cancer types, none have achieved global marketing approval. 17-AAG, which has entered clinical phase III, has shown poor efficacy, off-target effects, and intolerable adverse reactions, such as hepatotoxicity, ocular toxicity, and cardiac toxicity, leading to clinical trial terminations [56–59].

Given that HSP90 inhibitors encounter challenges during clinical development because of off-target effects and intolerable adverse reactions, the development of alternative therapeutic strategies is crucial. In contrast, polydatin, derived from traditional Chinese medicine with an uncomplicated structure, exhibits low toxicity and distinct advantages. These characteristics make it an attractive candidate for further development as a therapeutic agent. Notably, polydatin exhibited specificity by not interfering with upstream signaling events or the NF-κB pathway, underscoring its targeted action in promoting NLRP3 degradation. Furthermore, polydatin’s effectiveness in targeting different phases of NLRP3 inflammasome activation demonstrates its potential as a therapeutic option for diseases linked to inflammasome activity. The specific down-regulation of NLRP3 expression by polydatin, without affecting other components such as ASC, pro-CASP1, or NEK7, suggests a precise degradation mechanism. Importantly, polydatin demonstrates superior selectivity in inducing NLRP3 degradation, overcoming the limitations observed with MCC950 against self-activating mutants. HSP90α plays a critical role in stabilizing NLRP3 monomers and cage-like structures. Through its targeted action on HSP90α, polydatin disrupts the formation of cage-NLRP3 complexes, presenting a novel avenue for NLRP3 inflammasome regulation.

In conclusion, our study establishes a novel strategy for regulating the NLRP3 inflammasome by disrupting the structural integrity of the NLRP3 inflammasome complexes. Our findings demonstrate that polydatin, identified as a promising therapeutic agent, shows potential in treating AP and other inflammasome-related diseases by targeting different states of the NLRP3 inflammasome complexes. Further studies are warranted to fully elucidate the clinical potential of polydatin and to optimize its therapeutic application in inflammatory disorders.

## Materials and Methods

### Mice

C57BL/6J mice were purchased from GemPharmatech Co. Ltd. (Nanjing, China). Male mice matched for age (6 to 10 weeks) were utilized in this study. The macrophage-specific NLRP3-KO (NLRP3^lyz−/−^) mice were generated by crossing NLRP3 flox/flox mice with Lyz2-Cre transgenic mice. The animal welfare and experimental procedures were approved by the Institutional Animal Ethics and Welfare Committee of Nanjing University (Nanjing, China). All steps were taken to minimize animal suffering and reduce the number of animals used in the study.

### Chemicals, reagents, and antibodies.

Macrophage colony-stimulating factor (M-CSF) (81627-83-0) was purchased from PeproTech (Rocky Hill, NJ, USA). LPS (Escherichia coli 0111: B4, L3024), ATP (A7699), nigericin (72445), and uric acid sodium (U2875) were purchased from Sigma (St. Louis, MO, USA). Cerulein (Pep03263) was purchased from NJPeptide (Nanjing, China). GA (T6343) and THL (T67708) were purchased from TargetMol (Shanghai, China). Enzyme-linked immunosorbent assay (ELISA) kits for murine TNF-α, IL-1β, and human IL-1β were purchased from Dakewe Biotech Co. Ltd. (Beijing, China). FAM-FLICA caspase-1 assay KIT was purchased from Immunochemistry Technologies. Lipase, amylase, and MPO activity assay kits were purchased from the Nanjing Jiancheng Bioengineering Institute (Nanjing, China). The Liposomal Transfection Reagent (40802ES02) was purchased from Yeasen (Shanghai, China). Anti-NLRP3 (D4D8T, 1:2,000 dilution), anti-ASC (D2W8U, 1:2,000 dilution), anti-IL-1β (D3U3E, 1:2,000 dilution), and anti-LC3B (E7X4S, 1:2,000 dilution) were purchased from Cell Signaling Technology (Danvers, MA, USA). Anti-NEK7 (ERP4900, 1:2,000 dilution) was purchased from Abcam (Cambridge, UK). Anti-CASP1 (AG-20B-0042, 1:2,000 dilution) was purchased from Adipogen (San Diego, CA, USA). Anti-β-actin (M20010, 1:2,000 dilution), anti-β-tubulin (M20005, 1:2,000 dilution), anti-GAPDH (glyceraldehyde-3-phosphate dehydrogenase) (M20006, 1:2,000 dilution), anti-GFP-tag (M20004, 1:2,000 dilution), anti-DYKDDDDK-tag (M20008, 1:2,000 dilution), and anti-HA-tag (M10004, 1:2,000 dilution) were purchased from Abmart (Shanghai, China). Anti-HSP90α (A13501, 1:1,000 dilution) was purchased from the ABclonal Biotechnology (Wuhan, China). Anti-Tom20 (sc-136211, 1:2,000 dilution) was purchased from Santa Cruz Biotechnology (Santa Cruz, CA, USA). Alexa Fluor 488 goat anti-rabbit immunoglobulin G (IgG) (A11008) and Alexa Fluor 594 goat anti-mouse IgG (A11032) were purchased from Thermo Fisher Scientific (Waltham, MA, USA). Anti-Cbl-b (A2014) was purchased from ABclonal (Wuhan, China).

### AP model

The mice were fasted for over 12 h (water was given ad libitum) before the induction of pancreatitis by cerulein. Cerulein (100 μg/kg, intraperitoneally) was injected into the mice hourly for 7 injections. The mice were sacrificed 30 min after the last injection [60]. The mice were randomly divided into the AP group and 5, 10, and 20 mg/kg polydatin groups. Littermates injected with saline were used as controls (6 mice per group). Polydatin was administered 1 h before the first cerulein injection.

### MSU-induced peritonitis

Male C57BL/6 mice (18 to 20 g) received intraperitoneal injections of polydatin at a dose of 20 mg/kg once daily for 3 d, followed by an intraperitoneal injection of 2 mg/ml MSU. Six hours after injection, the mice were euthanized, and the peritoneal cavities were flushed with 3 ml of phosphate-buffered saline (PBS).

### Cell preparation

Bone marrow-derived macrophages and NLRP3^lyz−/−^ BMDMs were differentiated with M-CSF (25 ng/ml) for 5 to 6 d. HEK293T cells were cultured in Dulbecco’s modified Eagle’s medium (DMEM) supplemented with 10% fetal bovine serum (FBS). THP-1 cells were cultured in RPMI 1640 supplemented with 10% FBS.

### Depletion and reconstitution of macrophages in mice

Macrophages were pharmacologically depleted using clodronate liposomes as previously described [61]*.* Briefly, 100 μl of clodronate liposomes or control liposomes was intraperitoneally injected into 8-week-old male C57BL/6J mice to deplete macrophages. Twenty-four hours later, 2 × 10^6^ HSP90α KO BMDMs were intravenously injected to reconstruct macrophages. Depletion and reconstitution of macrophages were analyzed by flow cytometry.

### Plasmids and transfection

Full-length human HSP90α with a C-terminal GFP tag or mCherry tag was constructed using the pFlag-CMV-3. Deletion mutants of human NLRP3 with a flag tag, full-length human NLRP3 with a flag tag or GFP tag, human NLRP3 (L353P) with a GFP tag, and the ASC PYD plasmid were cloned into pcDNA3.1. The retroviral expression construct for mouse NLRP3 (L351P) was gifted by R.-H. Yin (Institute of Radiation Medicine, Beijing, China). HA-NEK7, GFP-ASC, pBiFC-NLRP3-VN173 (N-terminal), and pBiFC-NLRP3-VC155 (N-terminal) were synthesized by Generalbiol. HA-tagged ubiquitin, ubiquitin K48-only (forms only K48-linked chains), and ubiquitin K63-only (forms only K63-linked chains) plasmids were preserved in the laboratory. Transient transfections were performed using Lipofectamine 2000 (11668019; Invitrogen) and Hieff Trans Liposomal Transfection Reagent (40802ES03; Yeasen), according to the manufacturer’s instructions, in HEK293T cells.

### Reconstruction of the human inflammasome system in HEK293T cells

The human inflammasome system was reconstructed in HEK293T cells according to the previous publication [62]. Briefly, HEK293T cells were seeded at a density of 8 × 10^5^ cells/ml in 6-well plates. The following day, cells were transfected with plasmids encoding human GFP-NLRP3 L353P, GFP-ASC, flag-pro-CASP1, and HA-NEK7 using Lipofectamine 2000. Six hours later, the medium was refreshed with DMEM containing FBS (10%) and penicillin–streptomycin (1%). After 48 h, LPS was introduced to the cells to induce inflammasome activation.

### Quantitative real-time polymerase chain reaction

The mRNA levels of TLR4, IL-18, IL-1β, IL-6, NLRP3, and TNF-α were analyzed using quantitative real-time polymerase chain reaction (qRT-PCR) [63]. The murine primer sequences used for PCR were as follows: *β-actin*, 5′-GAGACCTTCAACACCCCAGC and 3′-ATGTCACGCACGATTTCCC; *IL-1β*, 5′-CTTCAGGCAGGCAGTATCACTC and 3′-TGCAGTTGTCTAATGGGAACGT; *IL-6*, 5′- ACAACCACGGCCTTCCCTAC and 3′-TCTCATTTCCACGATTTCCCAG; *TNF-α*, 5′-AGTGACAAGCCTGTAGCCC and 3′-AGGTTGACTTTCTCCTGGTAT; *TLR4*, 5′-TGCATGGATCAGAAACTCAGCAA and 3′- TGCCATGCCTTGTCTTCAATTGT; *NLRP3*, 5′-GAACTGCTGCCTCACTTCTAG and 3′-GGTGCTGGAGTGCCTCAC; *IL-18*, 5′-AATGTCTACCCTCTCCTGTAAG and 3′-CATCTTCCTTTTGGCAAGC. The mRNA levels were normalized to those of β-actin.

### FAM-FLICA and ELISA

The caspase-1 activity in cells and IL-1β and TNF-α in supernatants of the cell culture were analyzed according to the manufacturer’s instructions.

### Coimmunoprecipitation assay

The proteins were mixed with 1 μg of the corresponding antibody and then subjected to precipitation using protein A/G-agarose beads (Changzhou Smart-Lifesciences Biotechnology Co. Ltd). The immunoprecipitated proteins were separated using sodium dodecyl sulfate–polyacrylamide gel electrophoresis (SDS-PAGE), followed by immunoblotting using the appropriate antibodies.

### Immunofluorescence

BMDMs grown on confocal dish were stimulated and blocked with 5% goat serum at 4 °C for 2 h following incubation with anti-ASC, anti-CASP1, and anti-NLRP3 (1:100) overnight at 4 °C. After 3 rinses with 1× phosphate-buffered saline–Tween 20 (PBST), the sections were treated with a secondary fluorescent antibody (1:500) at room temperature for 2 h in the dark. After that, the nuclei were labeled with 4′,6-diamidino-2-phenylindole (DAPI) (Beyotime). All the cells were imaged using an inverted confocal microscope (Carl Zeiss, Germany).

For mouse tissues, paraffin-embedded sections of the mouse pancreas and lung were deparaffinized and rehydrated, followed by antigen retrieval using sodium citrate. Subsequently, the sections were blocked with 5% goat serum at 4 °C for 2 h. Anti-CASP1 p20 (1:100), anti-p-p65 (1:100), and anti-mouse F4/80 Dye-Ready monoclonal antibody were incubated overnight at 4 °C, and then the indicated steps were followed.

### ASC oligomerization assay

The ASC oligomerization assay was performed as previously reported [64].

### Blue native PAGE

BMDMs were first rinsed with cold PBS and then subjected to lysis in ice-cold native lysis buffer [500 mM ε-aminocaproic acid, 20 mM bis–tris, 10% (w/v) glycerol, 20 mM NaCl, 0.5% digitonin, 1 mM phenylmethylsulfonyl fluoride, 0.5 mM Na_3_VO_4_, 1× EDTA-free protease inhibitor, 0.5 mM NaF, and phosphatase inhibitor cocktail, pH 7.0] for 20 min on ice. Cell lysates were centrifuged at 16,000*g* for 15 min at 4 °C. Afterward, the samples were mixed with 5% G-250 sample additive and separated by 4 to 16% blue native PAGE using the Native PAGE Bis-Tris Gel System (Thermo Fisher Scientific).

### Cellular thermal shift assay

BMDMs were treated with either dimethyl sulfoxide (DMSO) or 10 μM polydatin for 3 h. Subsequently, the cells were harvested and underwent CETSA [65]. For the in vivo CETSA, C57BL/6 mice were treated with 20 mg/kg polydatin once daily for 3 consecutive days. Peritoneal macrophages were harvested and underwent CETSA.

### HSP90α expression and purification

HSP90α expression and purification were performed according to the method reported by Noddings et al. [66]*.*

### Microscale thermophoresis

Cell lysates containing HSP90-EGFP (enhanced GFP) or HSP90α protein were used. Fluorescence intensity was detected using the Nano Blue channel or Pico Red channel. The compound was serially diluted to create 16 distinct concentrations, mixed with the protein, and detected by capillary aspiration. The Affinity Analysis software was employed for the combined data analysis.

### Isothermal titration calorimetry

Proteins were purified using a buffer solution consisting of 30 mM Hepes (pH 7.5), 10% glycerol, 50 mM KCl, 0.5% DMSO, and 2 mM tris(2-carboxyethyl)phosphine (TCEP). Polydatin was dissolved in the same buffer. ITC experiments were conducted at 25 °C using a Nano ITC instrument (TA Instruments). The titrations were performed by injecting 1-μl aliquots of polydatin (500 μM) into a calorimeter cell containing a 300-μl solution of HSP90α (50 μM) while maintaining a constant stirring. The raw heat change was baseline corrected, followed by analysis in Origin. The dissociation constant *K*_d_ = 1/*K*_a_ was calculated by combining constants.

### HSP90α ATPase activity assay

HSP90α ATPase activity was measured according to the manufacturer’s instructions (Ultra-trace total ATPase test kit, A070-1-2).

### Statistical analysis

The results are presented as the mean ± SEM of 3 independent experiments, with each experiment comprising triplicate sets. Statistical analysis was performed using one-way analysis of variance (ANOVA) followed by Dunnett’s test to compare the control group with the multiple-dose groups. A significance level of *P* < 0.05 was considered statistically significant.
