# Supplementary material for: Polydatin-Mediated Inhibition of HSP90α Disrupts NLRP3 Complexes and Alleviates Acute Pancreatitis
Source: Research (Wash D C). 2024 Dec 17;7:0551. doi: 10.34133/research.0551 (PMC11651664; doi:10.34133/research.0551)
Supplement: Supplementary 1 — Figs. S1 to S13 [file research.0551.f1.zip › Supplementary fig 2024-11-15.docx]

**
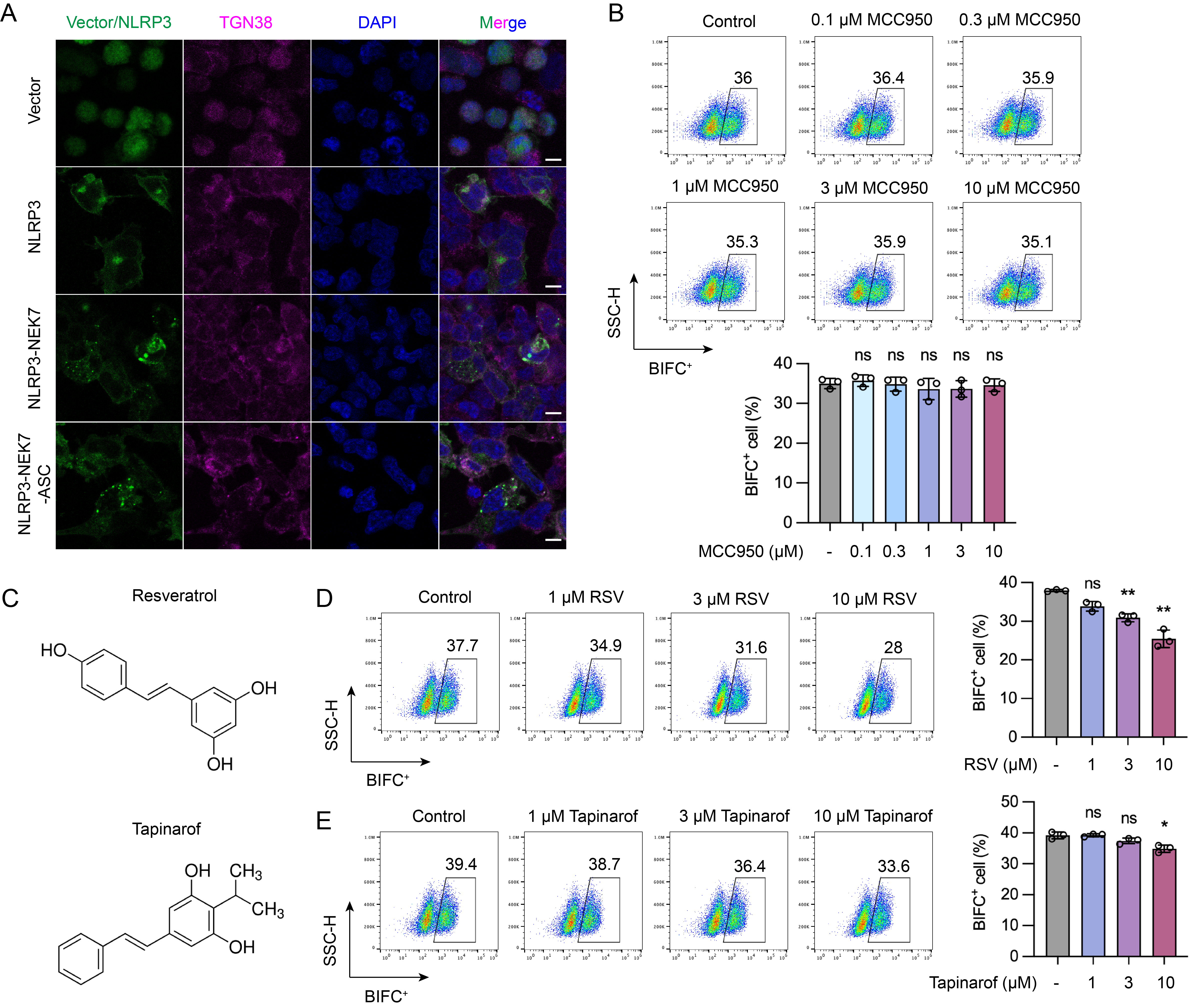
**

**Supplementary fig. 1 Effects of polydatin analogs on pre-formed cage-NLRP3.** (A) Cage-NLRP3, NLRP3-NEK7 complex and NLRP3-NEK7-ASC complex were reproduced in HEK293T cells. For cage-NLRP3, HEK293T cells were transfected with GFP-NLRP3 only; For NLRP3-NEK7 complex, HEK293T cells co-transfected with GFP-NLRP3 and HA-NEK7 were stimulated with nigericin, then ATPγS and MgCl_2_ were added to lock NLRP3 in an active conformation. For NLRP3-NEK7-ASC complex, HEK293T cells co-transfected with GFP-NLRP3, HA-NEK7 and ASC^PYD^ were stimulated with nigericin, then ATPγS and MgCl_2_ were added. Cage-NLRP3, NEK7-NLRP3 complex and NEK7-NLRP3-ASC complex were observed by Confocal microscopy. (B) HEK293T cells co-transfected with BIFC-nYFP-NLRP3 and BIFC-cYFP-NLRP3 were treated with indicated concentrations of MCC950, and the BIFC^+^ cells were determined by flow cytometry. (C) Chemical structure of Tapinarof and Resveratrol. (D-E) HEK293T cells co-transfected with BIFC-nYFP-NLRP3 and BIFC-cYFP-NLRP3 were treated with indicated concentrations of (D) Resveratrol or (E) Tapinarof, and the BIFC^+^ cells were determined by flow cytometry. Scale bar: 10 μm. Data are presented as mean ± SEM of three independent experiments. * *P* < 0.05, ** *P* < 0.01 vs. as indicated. RSV: Resveratrol.


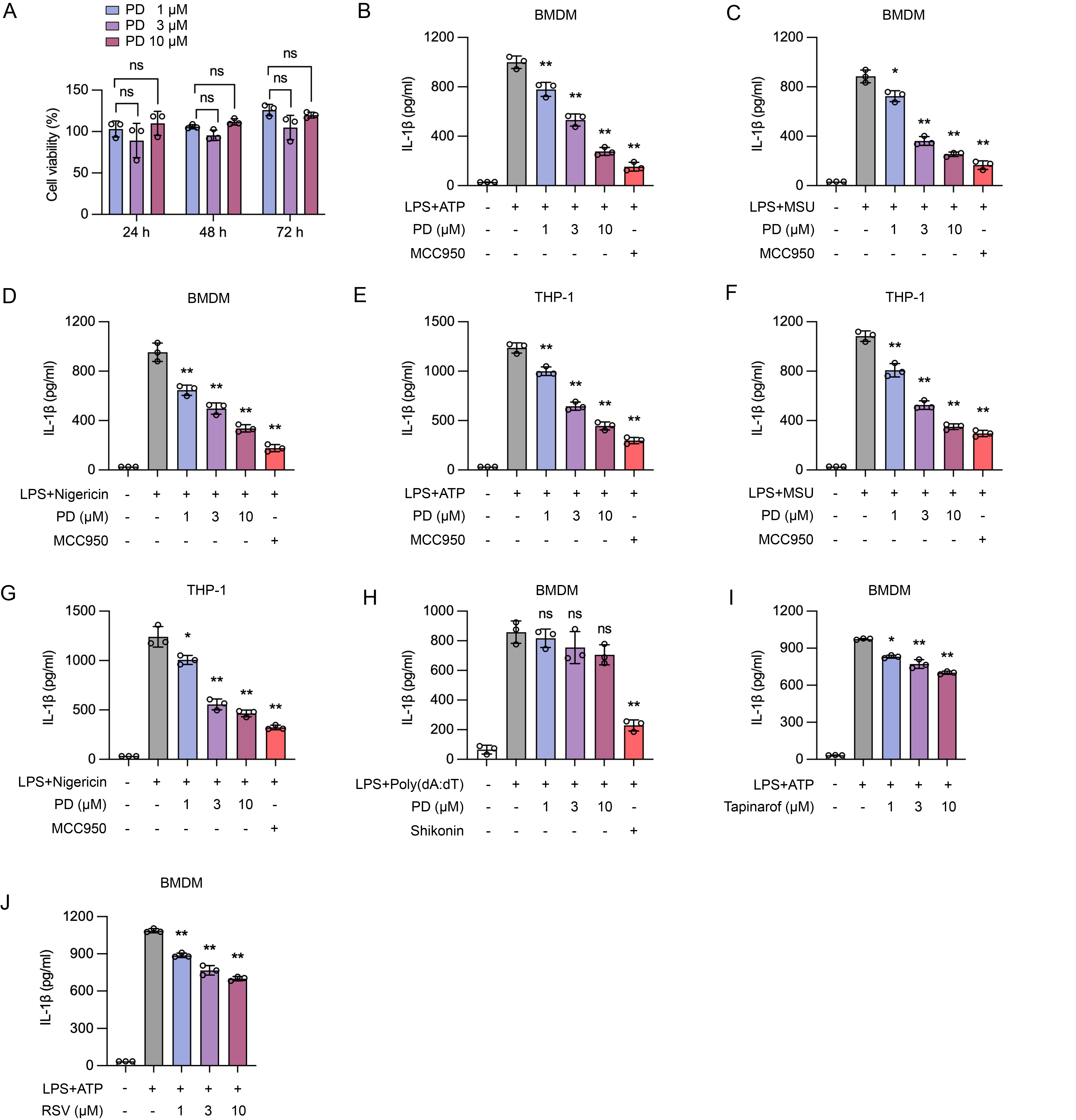


**Supplementary fig. 2 Polydatin selectively inhibits IL-1β secretion.** (A) BMDMs were treated with Polydatin for the indicated concentrations for 24 h, 48 h and 72 h and then cell viability was determined by MTT assay. (B-D) BMDMs were stimulated with 100 ng/ml LPS for 3 h, followed by indicated concentrations of Polydatin treatment for 1 h and then another 1 h of 5 mM ATP (B), 2 h of 500 μg/ml MSU (C) or 2 h of 10 μM Nigericin stimulation (D). IL-1β in supernatant were determined by ELISA. (E-G) PMA (10 ng/ml)-differentiated THP-1 cells were stimulated with 100 ng/ml LPS for 3 h, followed by indicated concentrations of Polydatin treatment for 1 h and then another 1 h of 5 mM ATP (E), 2 h of 500 μg/mL MSU (F) or 2 h of 10 μM Nigericin stimulation (G). IL-1β in supernatant were determined by ELISA. (H) BMDMs were stimulated with 100 ng/ml LPS for 3 h, followed by indicated concentrations of Polydatin treatment for 1 h and then overnight poly(dA:dT) stimulation. IL-1β in supernatant were determined by ELISA. (I-J) BMDMs were stimulated with 100 ng/ml LPS for 3 h, followed by indicated concentrations of Tapinarof (I) or Resveratrol (J) treatment for 1 h and then another 1 h of 5 mM ATP. IL-1β in supernatant were determined by ELISA. Data are presented as mean ± SEM of three independent experiments. * *P* < 0.05, ** *P* < 0.01 vs. LPS + ATP/MSU/Nigericin/ poly(dA:dT). PD: Polydatin. RSV: Resveratrol.


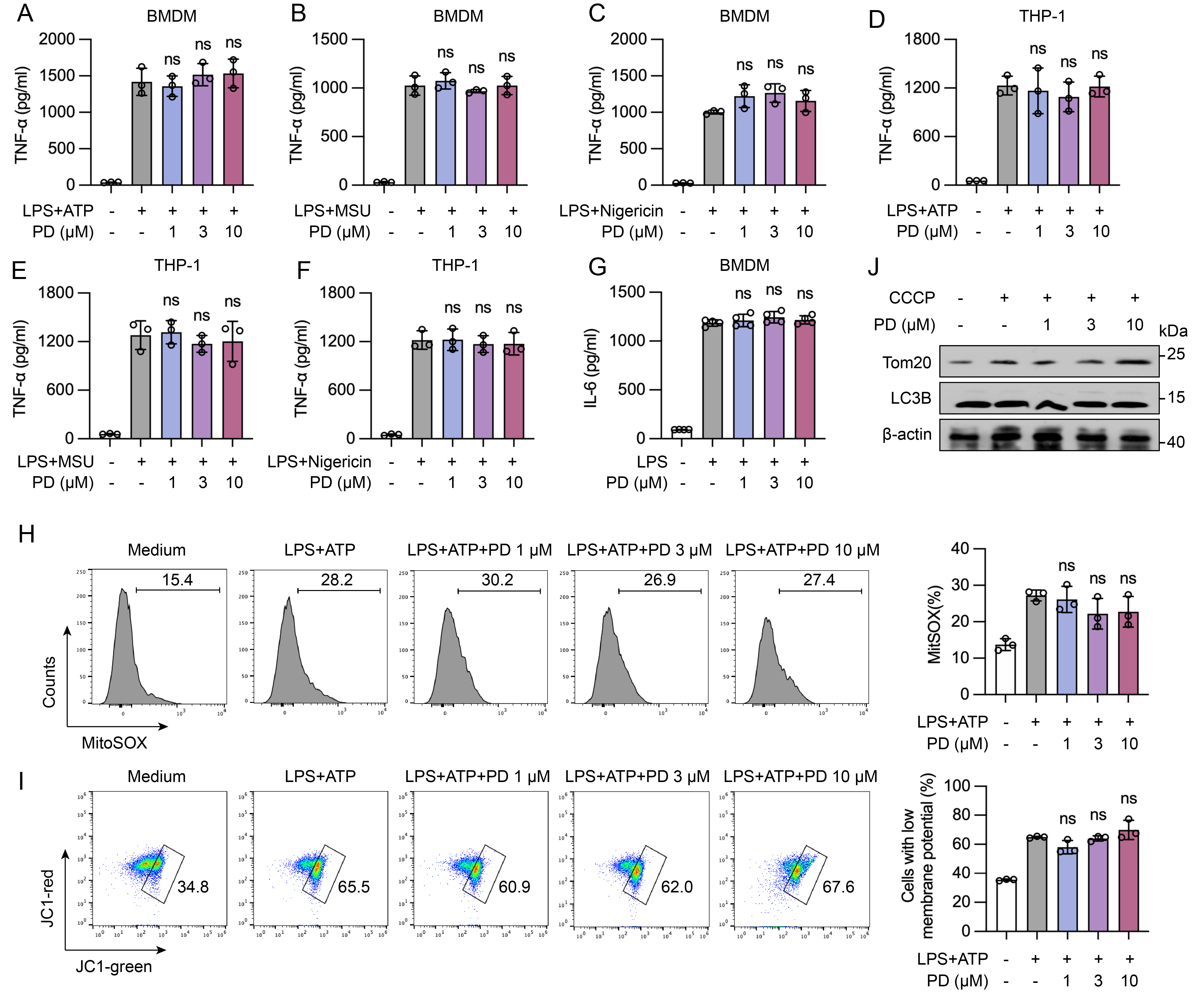


**Supplementary fig. 3. Polydatin has no effect on the secretion of TNF-α or IL-6 and mitochondrion damage.** (A-C) BMDMs were treated with indicated concentrations of Polydatin, then stimulated with 100 ng/ml LPS for 3 h and 1 h of 5 mM ATP (A), 2 h of 500 μg/mL MSU (B) or 2 h of 10 μM Nigericin stimulation (C). TNF-α in supernatant were determined by ELISA. (D-F) PMA (10 ng/ml)-differentiated THP-1 were treated with indicated concentrations of Polydatin, then stimulated with 100 ng/ml LPS for 3 h and 1 h of 5 mM ATP (D), 2 h of 500 μg/ml MSU (E) or 2 h of 10 μM Nigericin stimulation (F). TNF-α in supernatant were determined by ELISA. (G) BMDMs were treated with indicated concentrations of Polydatin, then stimulated with 100 ng/ml LPS for 3 h. IL-6 in supernatant were determined by ELISA. (H) LPS-primed BMDMs were treated with Polydatin (1, 3 or 10 μM) for 1 h and then left stimulated with 5 mM ATP for 1 h, followed by staining with MitoSOX or (I) JC-1 to determine the ROS level and mitochondrial membrane potential. (J) BMDMs were treater with indicated concentrations of Polydatin and then left stimulated with CCCP for 18 h. The level of Tom20 and LC3B were determined by immunoblot. Data are presented as mean ± SEM of three independent experiments. PD: Polydatin.


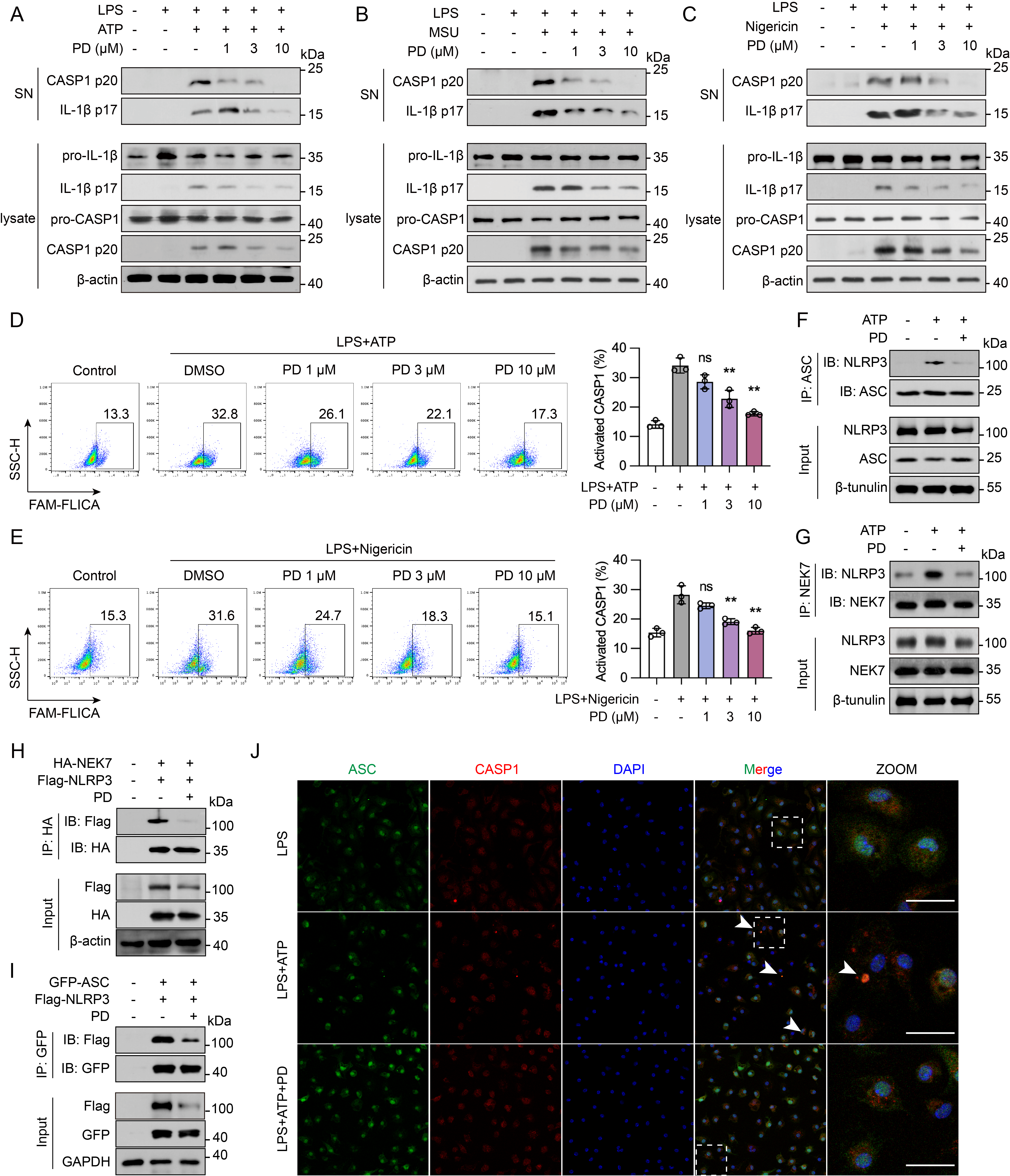


**Supplementary fig. 4. Polydatin inhibits NLRP3 inﬂammasome assemblyand CASP1 activation.** (A-C) BMDMs were stimulated with 100 ng/ml LPS for 3 h, followed by indicated concentrations of Polydatin treatment for 1 h and then another 1 h of 5 mM ATP (A), 2 h of 500 μg/ml MSU (B) or 2 h of 10 μM Nigericin (C) stimulation. Cleaved IL-1β (p17), activated CASP1 (p20) in culture supernatants and pro-IL-1β, IL-1β p17, pro-CASP1, CASP1 p20 in lysates of BMDMs were analyzed by immunoblot. (D-E) BMDMs were stimulated with 100 ng/ml LPS for 3 h, followed by indicated concentrations of Polydatin treatment for 1 h and then another 1 h of 5 mM ATP (D) or 2 h of 10 μM Nigericin (E) stimulation. CASP1 activation was determined by FAM-FLICA staining. (F-G) An endogenous immunoprecipitation (IP) was performed using (F) ASC antibody or (G) NEK7 antibody in LPS-primed BMDMs stimulated with ATP, with or without Polydatin. (H-I) HEK293T cells were transfected with HA-NEK7 and flag-NLRP3 or GFP-ASC in the presence or absence of Polydatin. (H) IP and western blotting analysis of NLRP3-NEK7 interaction. (I) IP and western blotting analysis of NLRP3-ASC interaction. (J) LPS primed BMDMs were treated with 10 μM Polydatin for 1 h, followed by ATP stimulation for 30 min. Co-localization of ASC and pro-CASP1 was determined by immunofluorescence. Scale bar 10 μm. Data are presented as mean ± SEM of three independent experiments. * *P* < 0.05, ** *P* < 0.01 vs. LPS + ATP/MSU/Nigericin. SN: supernatants. PD: Polydatin.


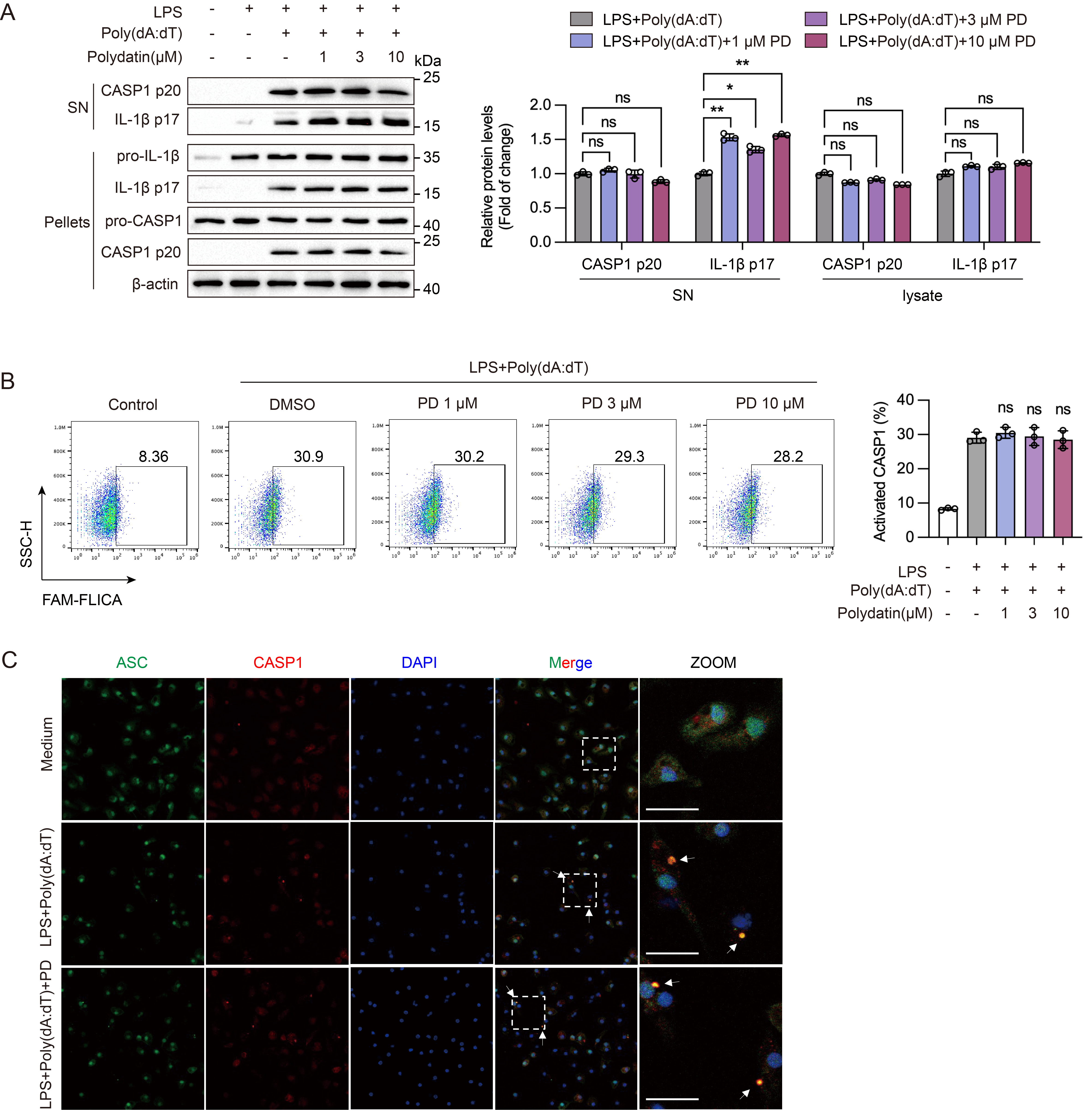


**Supplementary fig. 5 Polydatin has no effect on the activation of AIM2 inflammasome in macrophages.** (A) BMDMs were stimulated with 100 ng/ml LPS for 3 h, followed by indicated concentrations of Polydatin treatment for 1 h and then another 6 h of poly(dA:dT) (1 μg/10^6^ cells). Cleaved IL-1β (p17), activated CASP1 (p20) in culture supernatants and pro-IL-1β, IL-1β p17, pro-CASP1, CASP1 p20 in lysates of BMDMs were analyzed by immunoblot. (B) CASP1 activation was determined by FAM-FLICA staining. (C) LPS primed BMDMs were treated with Polydatin (10 μM) for 1 h, followed by poly(dA:dT) (1 μg/10^6^ cells) stimulation for 6 h. Co-localization of ASC and pro-CASP1 was determined by immunofluorescence. Scale bar 10 μm. PD: Polydatin. Data are presented as mean ± SEM of three independent experiments. * *P* < 0.05, ** *P* < 0.01 vs. LPS + ATP/MSU/Nigericin. PD: Polydatin.


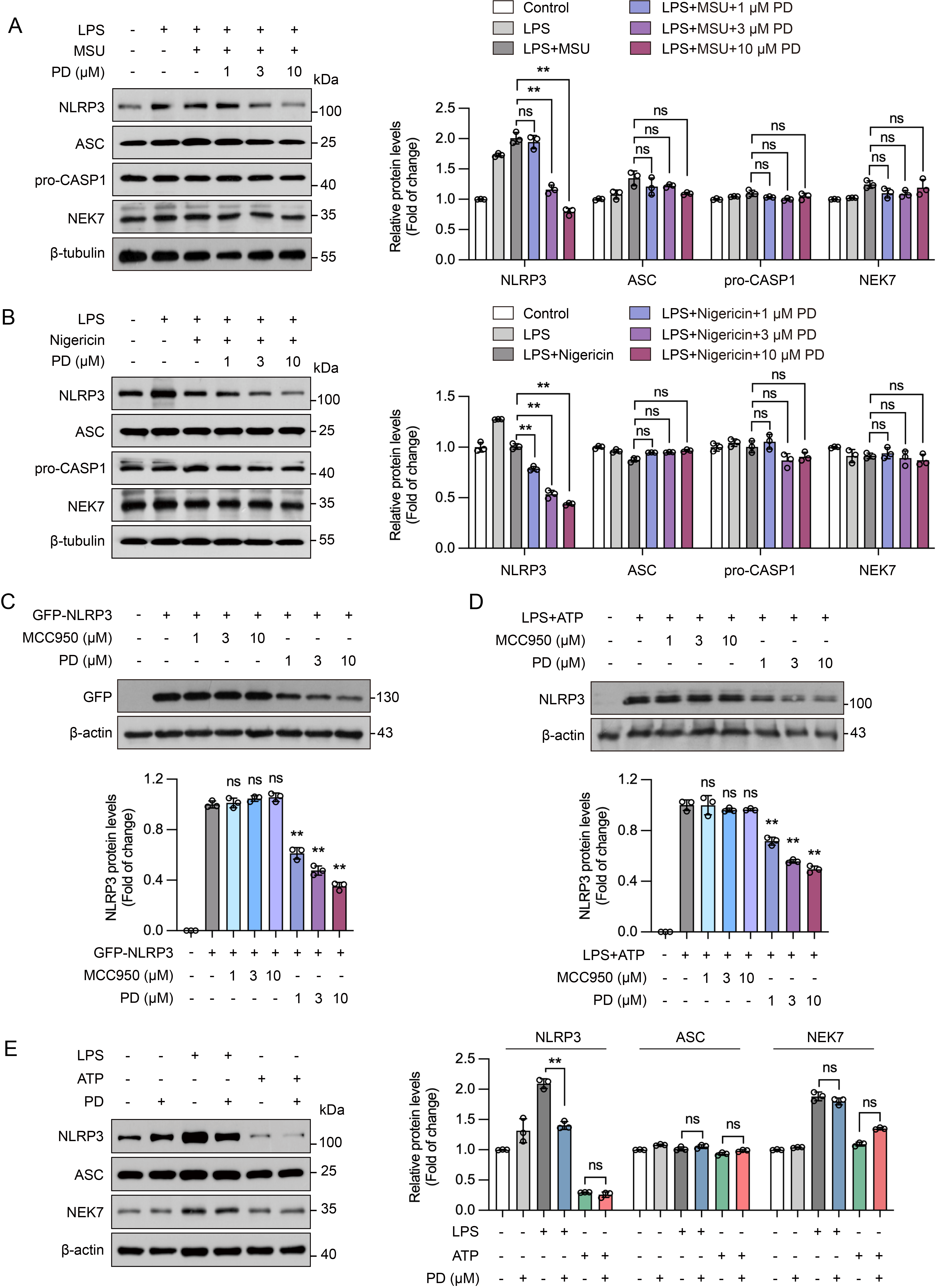


**Supplementary fig. 6 Polydatin specifically inhibits the expression of NLRP3.** (A-B) BMDMs were stimulated with 100 ng/ml LPS for 3 h, followed by indicated concentrations of Polydatin treatment for 1 h and then another 2 h of 500 μg/mL MSU (A) or 2 h of 10 μM Nigericin (B). Western blot analysis of NLRP3, ASC, NEK7 and pro-CASP1. (C) HEK293T cells were transfected with GFP-NLRP3 and then treated with indicated concentrations of Polydatin or MCC950 treatment for 1 h. The level of GFP-NLRP3 was determined by western blot. (D) PMA (10 ng/ml)-differentiated THP-1 cells were stimulated with 100 ng/ml LPS for 3 h, followed by indicated concentrations of Polydatin or MCC950 treatment for 1 h and then another 1 h of 5 mM ATP. The level of NLRP3 was determined by western blot. (E) BMDMs were treated with 10 μM Polydatin for 1 h before LPS or ATP treatment. Western blot analysis of NLRP3, ASC and NEK7. Data are presented as mean ± SEM of three independent experiments. ** P < 0.01. PD: Polydatin.


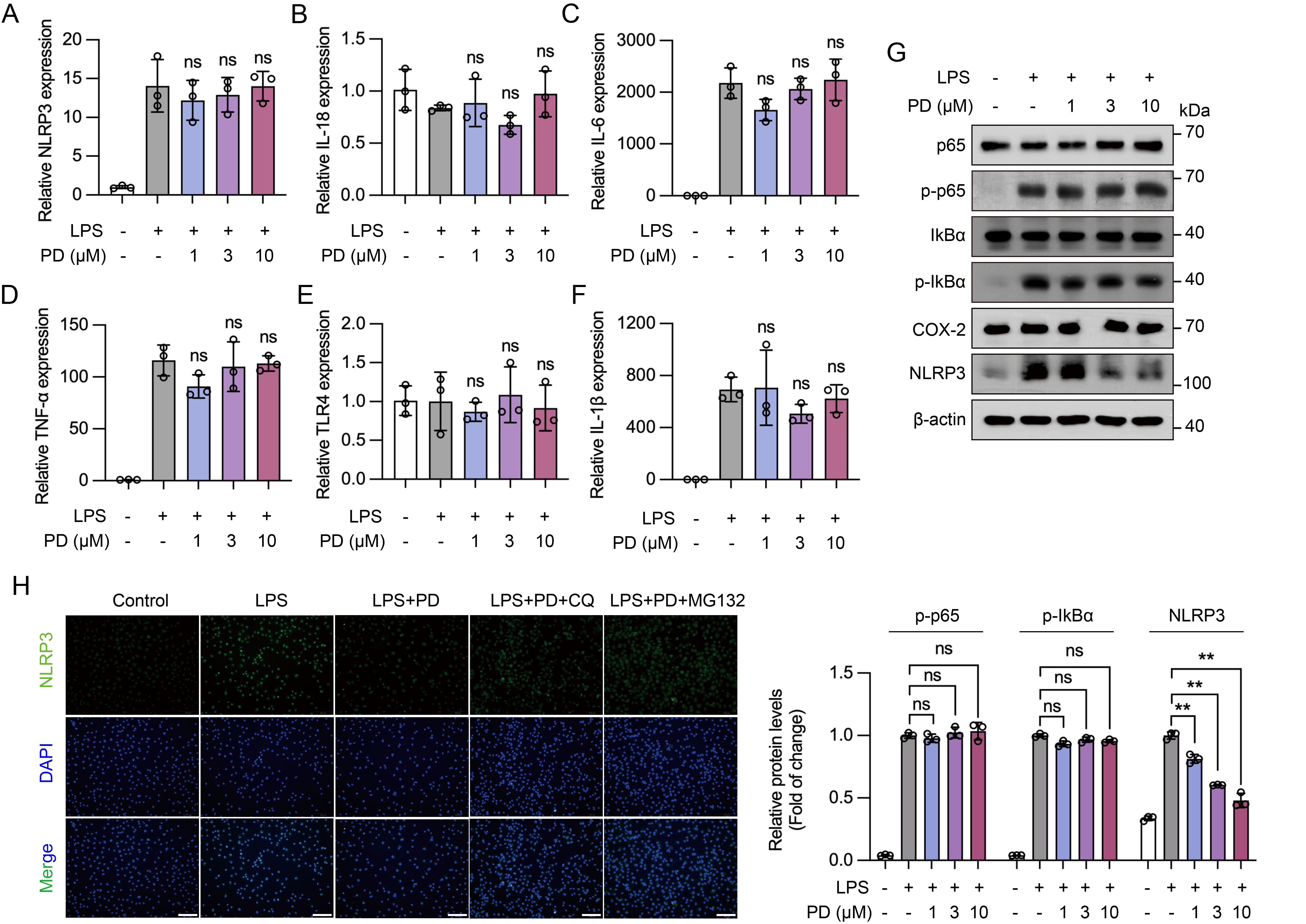


**Supplementary fig. 7 Polydatin has no effect on the level of inflammasome-independent cytokine.** (A-G) BMDMs were treated with Polydatin (1, 3 and 10 μM) before LPS treatment. The mRNA level of (A) NLRP3, (B) IL-18, (C) IL-6, (D) TNF-α, (E) TLR4 and (F) IL-1β were determined by qRT-PCR. (G) Expression of p65, p-p65, IkBα, p-IkBα, COX-2 and NLRP3 were determined by immunoblot. (H) BMDMs were treated with 20 μM MG132 or 30 μM CQ for 2 h, then 100 ng/ml LPS for 3 h followed by 10 μM Polydatin treatment for 1 h. The expression of NLRP3 was determined immunofluorescence. Scale bar 100 μm. Data are presented as mean ± SEM of three independent experiments. ** P < 0.01. PD: Polydatin.


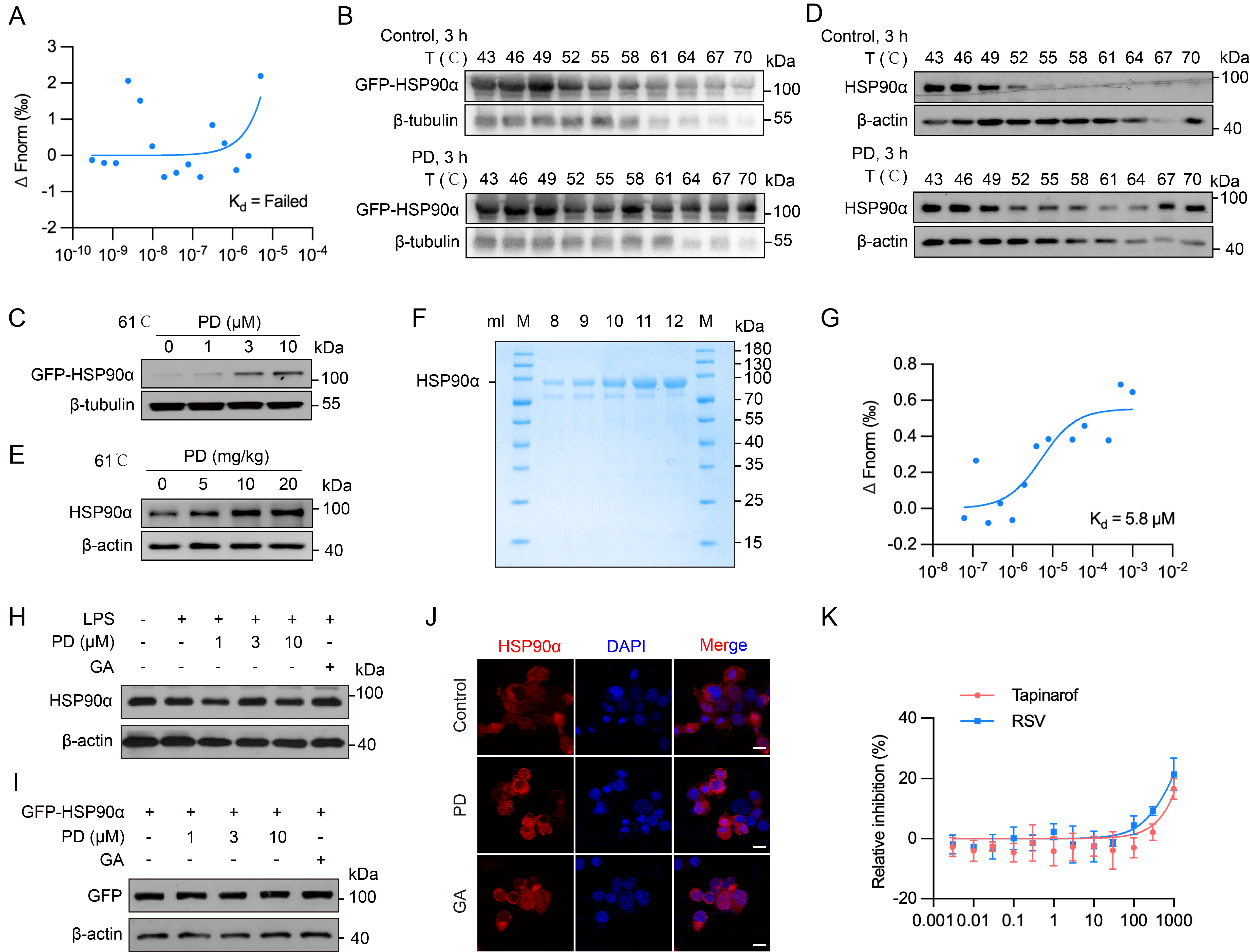


**Supplementary fig. 8 Polydatin directly binds to HSP90α without affecting its level.** (A) Cell lysate collected from HEK293T cells transfected with GFP-NLRP3 were used for MST assay for the affinity between Polydatin and NLRP3. (B) HEK293T cells transfected with GFP-HSP90α were treated with 10 μM Polydatin for 48 h and collected for CETSA. (C) HEK293T cells transfected with GFP-HSP90α were treated 1, 3 and 10 μM Polydatin for 48 h and collected for CETSA at 61 ℃. (D) Mice were i.g. with 20 mg/kg Polydatin once a day for three days. Peritoneal macrophages were collected and subjected for CETSA. (E) Mice were i.g. with 5, 10 and 20 mg/kg Polydatin once a day for three days. Peritoneal macrophages were collected and subjected for CETSA. (F) SDS-polyacrylamide gel electrophoresis (PAGE) of gel filtration fractions for the HSP90α. Locations of HSP90α was labeled. (G) Cell lysate collected from HEK293T cells transfected with GFP-HSP90α were used for MST assay for the affinity between Polydatin and HSP90α. (H) BMDMs were stimulated with 100 ng/ml LPS for 3 h, followed by indicated concentrations of Polydatin for 1 h. The level of HSP90α was analyzed by western blot. (I) HEK293T cells transfected with GFP-HSP90α were treated with indicated concentrations of Polydatin for 1 h. The level of HSP90α was analyzed by western blot. (J) The level of HSP90α in HEK293T cells was determined by Immunofluorescence. (K) Effect of Tapinarof and Resveratrol on the ATPase activity of HSP90α. After incubation HSP90α with indicated different concentrations of Tapinarof or Resveratrol, ATPase was measured by Ultra-trace total ATPase test kit. Scale bar: 10 μm. Data are presented as mean ± SEM of three independent experiments. PD: Polydatin. RSV: Resveratrol. GA: Geldanamycin.


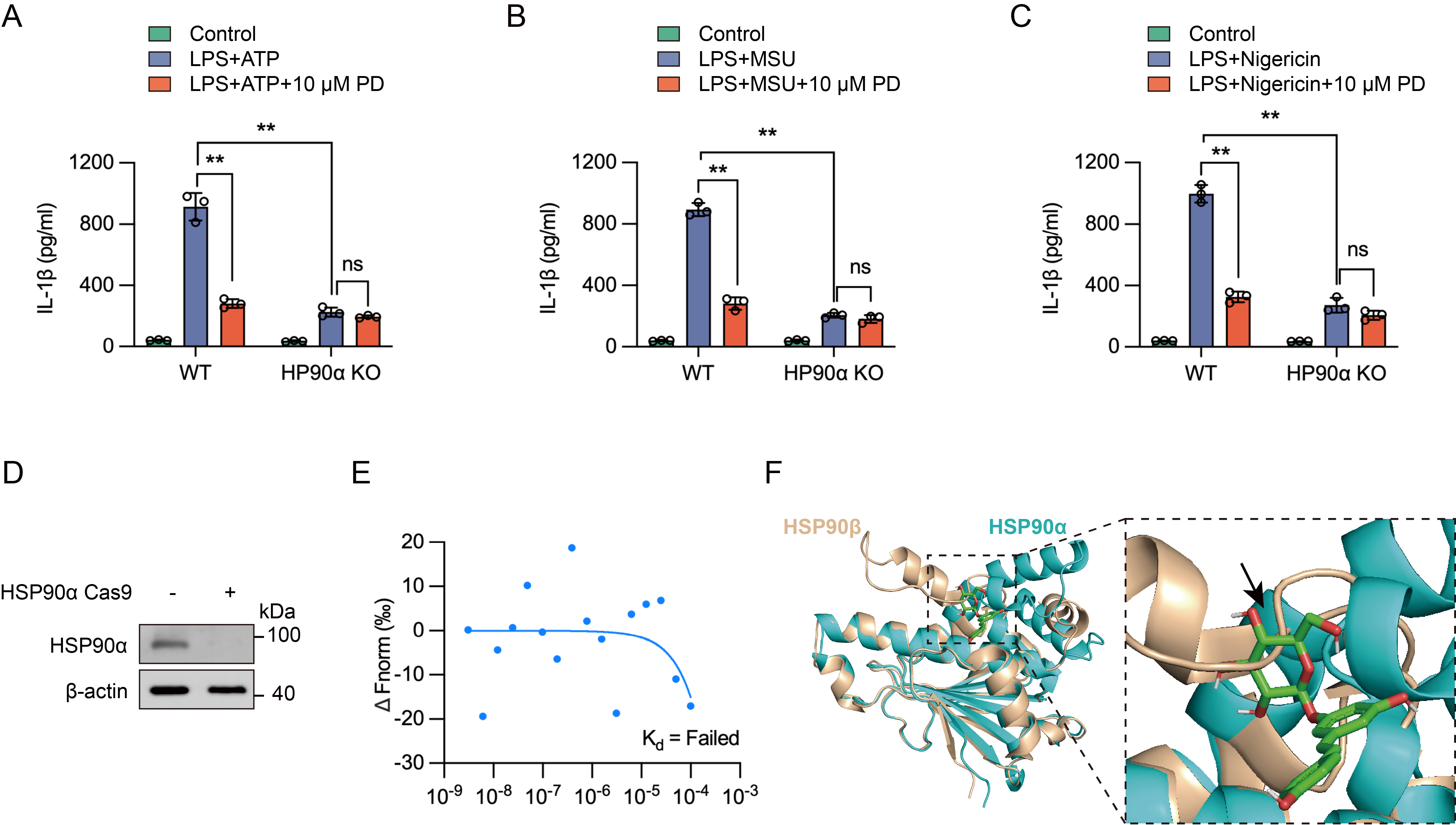


**Supplementary fig. 9 Polydatin selectively targets HSP90α rather than HSP90β to inhibit IL-1β secretion.** (A-C) BMDMs infected with HSP90α cas9 lentivirus were stimulated with 100 ng/ml LPS for 3 h, followed by 10 μM Polydatin for 1 h and then another 1 h of 5 mM ATP (A), 2 h of 500 μg/ml MSU (B) or 2 h of 10 μM Nigericin stimulation (C). IL-1β in supernatant were determined by ELISA. (D) After infecting BMDMs with HSP90α cas9 lentivirus, the expression of HSP90α was detected by immublot. (E) The affinity between Polydatin and recombinant HSP90β protein was assessed by MST assay. (F) Binding poses of Polydatin against HSP90α (Indigo, PDB ID: 5H22) and HSP90β (Beige, HSP90β PDB ID: 7Y04). Data are presented as mean ± SEM of three independent experiments. PD: Polydatin.


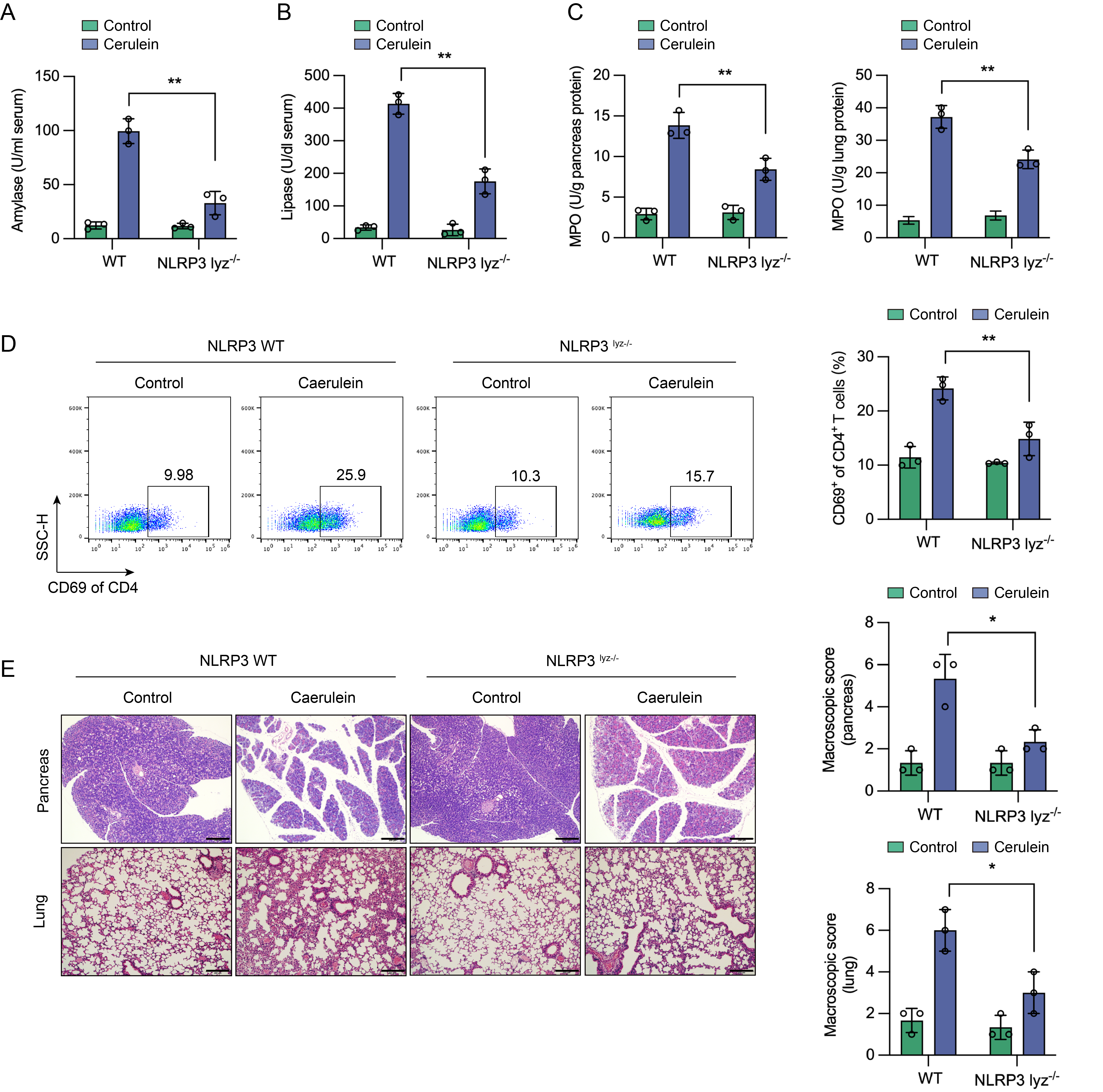


**Supplementary fig. 10 NLRP3 ^lyz-/-^ mice exhibit milder acute pancreatitis.** Acute pancreatitis was induced by cerulein stimulation in mice. (A) Serum amylase and (B) lipase activities were determined to reﬂect pancreatic damage and disease severity. (C) MPO level in lungs and pancreases were analyzed to reﬂect the severity of systemic inﬂammation. (D) Representative flow cytometry plots of CD4^+^ CD69^+^ T cells in spleen. (E) Representative H&E staining of mouse pancreas and lung tissues. Scale bar: 100 μm. Data are presented as mean ± SEM of 3 mice in each group. * *P* < 0.05, ** *P* < 0.01 vs. as indicated.


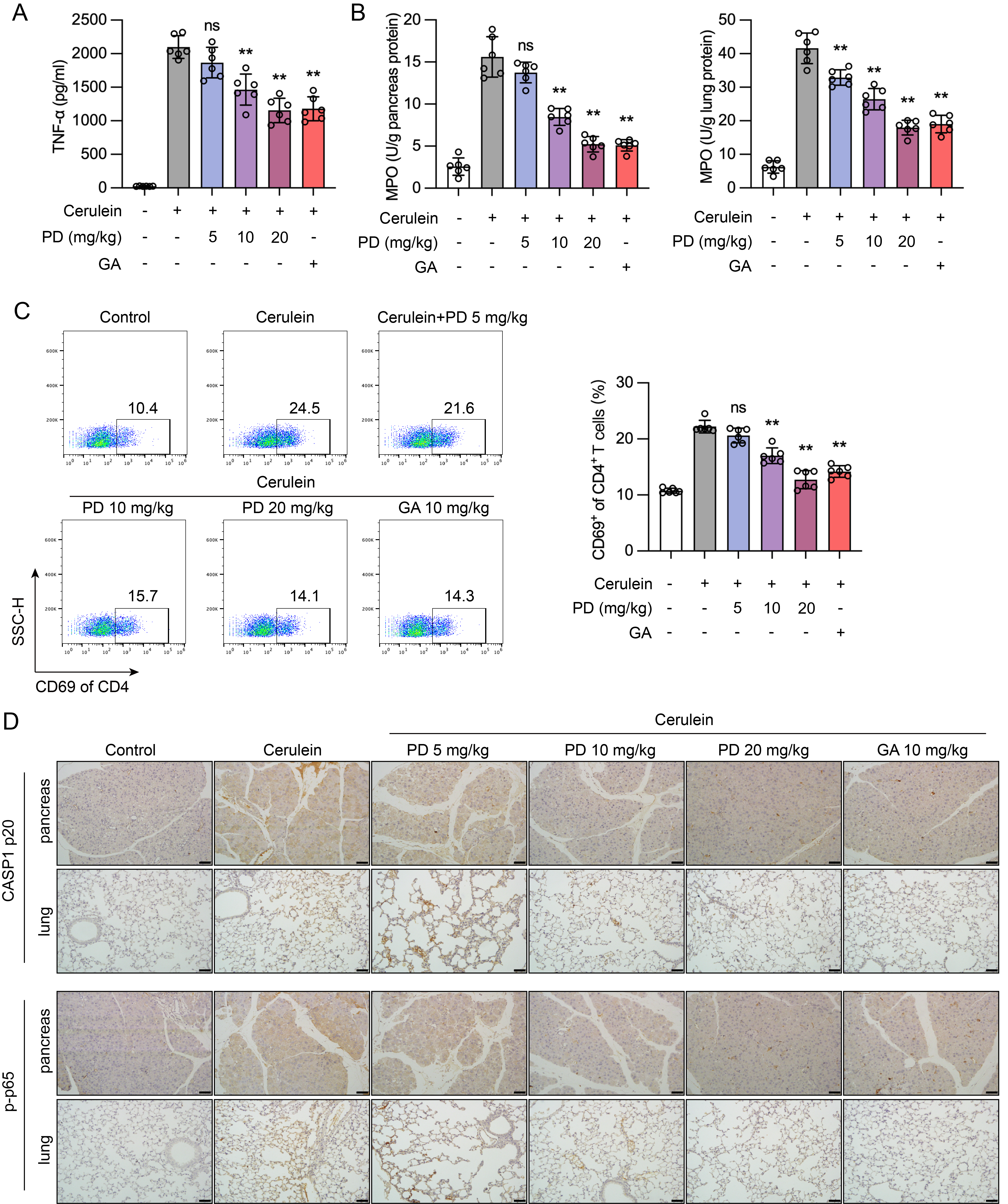


**Supplementary fig. 11 Polydatin decreases pancreas and lung damage in mice with cerulein-induced.** Acute pancreatitis was induced by cerulein stimulation in mice. Mice were treated with indicated concentrations of Polydatin or 10 mg/kg Geldanamycin (i.g.) before cerulein stimulation. (A) The level of TNF-α in serum was determined by ELISA. (B) MPO level in lungs and pancreases were analyzed to reﬂect the severity of systemic inﬂammation. (C) Representative flow cytometry plots of CD4^+^ CD69^+^ T cells in spleen. (D) Representative image of mouse pancreas and lung tissue sections for CASP1 p20 and p-p65 staining. Scale bar: 50 μm. Data are presented as mean ± SEM of 6 mice in each group. ** *P* < 0.01 vs. as indicated. PD: Polydatin. GA: Geldanamycin.


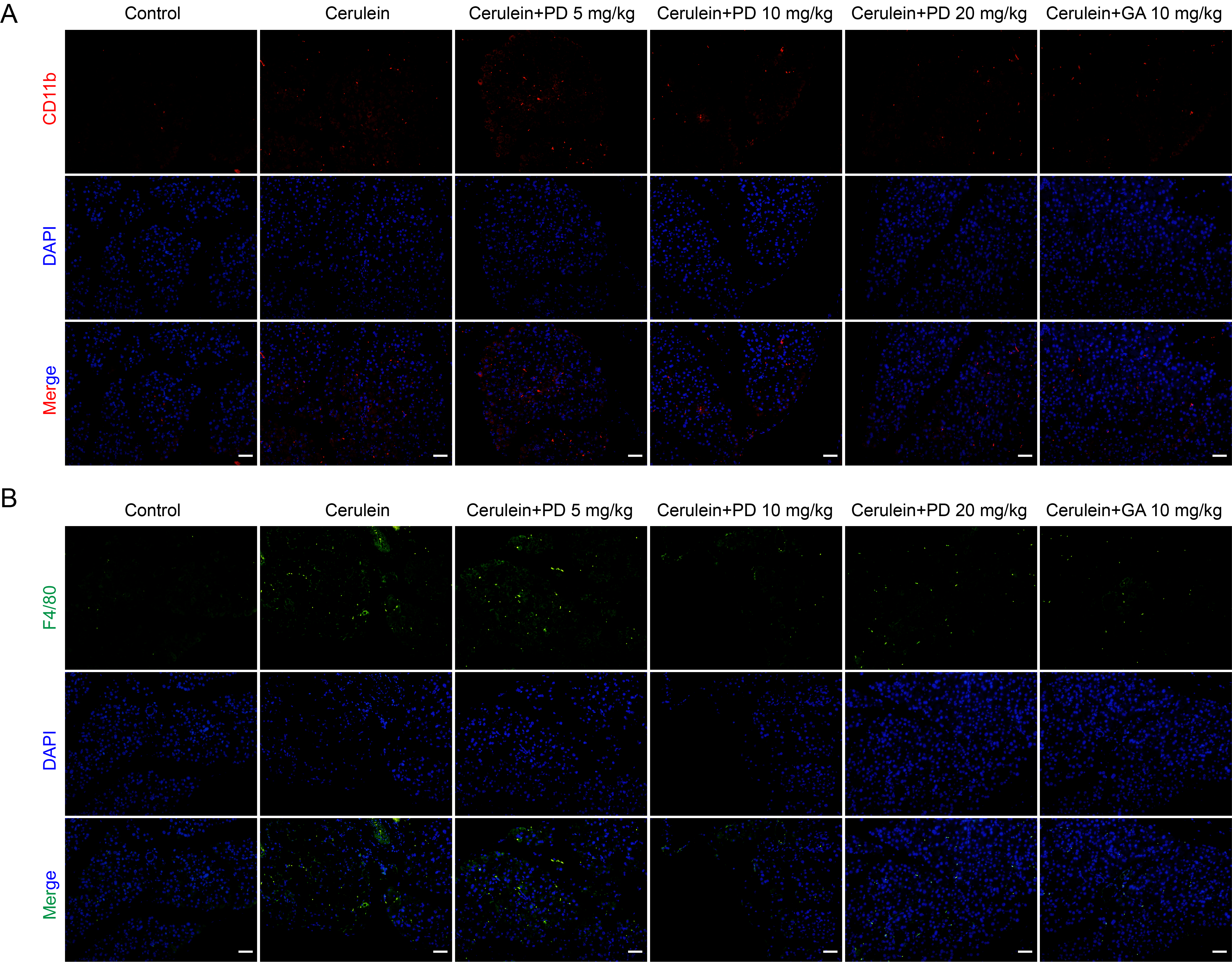


**Supplementary fig. 12 Polydatin inhibits the infiltration of inflammatory cells in pancreatic.** Acute pancreatitis was induced by cerulein stimulation in mice. Mice were treated with indicated doses of Polydatin (i.g.) before cerulein stimulation. (A) Immunofluorescence detection of CD11b and (B) F4/80 in the mouse pancreases. Scale bar: 50 μm. PD: Polydatin. GA: Geldanamycin.


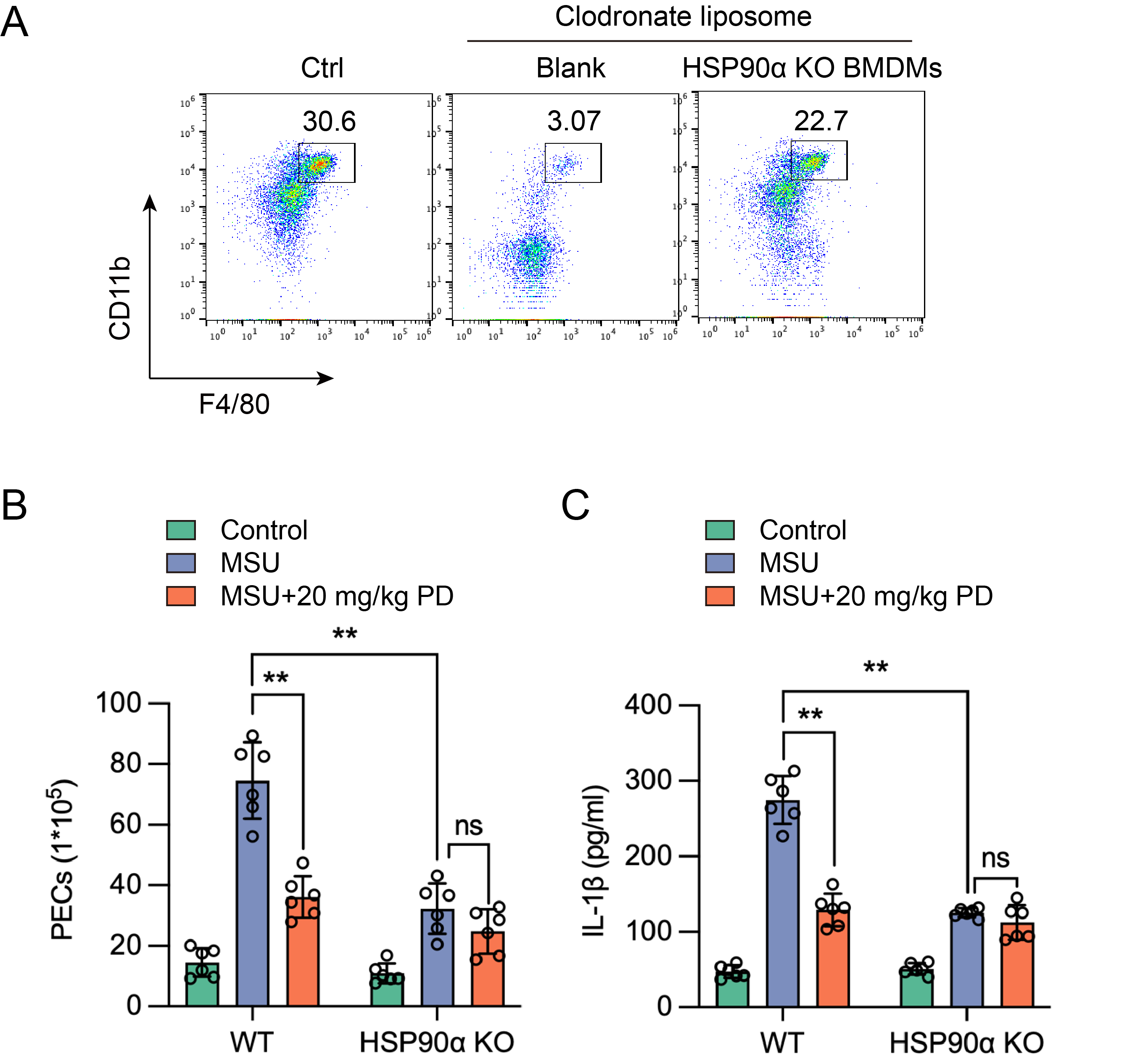


**Supplementary fig. 13 Recipient mice transplanted with HSP90α knockout BMDMs exhibit milder peritonitis.** (A) F4/80 and CD11b positive macrophages from peritoneal cavity were detected by flow cytometry. (B-C) Wild-type or HSP90α^-/-^ BMDMs-reconstituted mice were i.g. with 20 mg/kg Polydatin for 3 days (once a day) before i.p. injection of MSU (1 mg MSU crystals dissolved in 0.5 ml sterile PBS). After 6 h, the mice were euthanized, and the peritoneal cavities were lavaged with 3 ml cold PBS. (B) Peritoneal exudate cells (PECs) were collected and counted. (C) IL-1β in the peritoneal cavity was analyzed by ELISA. Data are presented as mean ± SEM of 6 mice in each group. * P < 0.05, ** P < 0.01 vs. as indicated. PD: Polydatin.
